# Estimating prevalence of human traits among populations from polygenic risk scores

**DOI:** 10.1186/s40246-021-00370-z

**Published:** 2021-12-13

**Authors:** Britney E. Graham, Brian Plotkin, Louis Muglia, Jason H. Moore, Scott M. Williams

**Affiliations:** 1 Departments of Population and Quantitative Health Sciences and Genetics and Genome Scenes, Cleveland Institute for Computational Biology, Case Western Reserve University, Cleveland, OH 44106 USA; 2 Systems Biology and Bioinformatics, Case Western Reserve University, Cleveland, OH 44106 USA; 3 Burroughs Wellcome Fund, Research Triangle Park, NC 27614 USA; 4 Cincinnati Children’s Hospital Medical Center, University of Cincinnati College of Medicine, Cincinnati, OH 45229 USA; 5 Department of Biostatistics, Epidemiology, and Informatics, Perelman School of Medicine, University of Pennsylvania, Philadelphia, PA 19104 USA

**Keywords:** Disease prevalence, PRS transferability, Universal risk variants, Genetic architecture

## Abstract

The genetic basis of phenotypic variation across populations has not been well explained for most traits. Several factors may cause disparities, from variation in environments to divergent population genetic structure. We hypothesized that a population-level polygenic risk score (PRS) can explain phenotypic variation among geographic populations based solely on risk allele frequencies. We applied a population-specific PRS (psPRS) to 26 populations from the 1000 Genomes to four phenotypes: lactase persistence (LP), melanoma, multiple sclerosis (MS) and height. Our models assumed additive genetic architecture among the polymorphisms in the psPRSs, as is convention. Linear psPRSs explained a significant proportion of trait variance ranging from 0.32 for height in men to 0.88 for melanoma. The best models for LP and height were linear, while those for melanoma and MS were nonlinear. As not all variants in a PRS may confer similar, or even any, risk among diverse populations, we also filtered out SNPs to assess whether variance explained was improved using psPRSs with fewer SNPs. Variance explained usually improved with fewer SNPs in the psPRS and was as high as 0.99 for height in men using only 548 of the initial 4208 SNPs. That reducing SNPs improves psPRSs performance may indicate that missing heritability is partially due to complex architecture that does not mandate additivity, undiscovered variants or spurious associations in the databases. We demonstrated that PRS-based analyses can be used across diverse populations and phenotypes for population prediction and that these comparisons can identify the universal risk variants.

**Supplementary Information:**

The online version contains supplementary material available at 10.1186/s40246-021-00370-z.

## Introduction

The prevalence of many phenotypes differs across populations. The causes of population variability, though not always well understood, can be partially due to different frequencies of common causative alleles that are shared among populations and/or variation in environmental exposure across these same populations. However, it is also possible that population-specific alleles affect prevalence. One way to increase our understanding of a trait’s genetic architecture and population differences in disease prevalences is to determine if variants associated with risk in one or a few populations can be extrapolated to the phenotypic burden for other populations across the world. For example, some variants that are extremely common in some populations are very rare in others despite having large phenotypic effects [26]. It has been suggested that most heritability can be explained by variants associated with a specific phenotype that are not in the group of “core” variants thought to affect trait characteristics [3]. These “core” variants may, however, not necessarily be those determined to be most statistically associated, although there may be overlap. Examining variants that do or do not transfer among populations may help elucidate the concept of “core” genes.

Models of genetic architecture often assume that the effects of a trait’s genetic components are additive, without interaction, and highly similar across populations. The assumption of additivity disregards potential complexity, but can be implicitly tested by assessing how well a genetic model explains the genotype to phenotype relationship [17].

One additive model used to predict phenotypic status is the polygenic risk score (PRS), that has, in general, been used to elucidate an individual’s risk of a specific phenotype [27, 61]. A PRS for a disease is usually calculated as the sum of an individual’s risk associated alleles, sometimes weighted by the average effects of these alleles.

Commonly, calculated individual PRSs also assume that a trait’s genetic architecture is additive and that neither gene–gene nor gene-environment interactions are important factors. This method has seen some success, but often fails to predict an individual’s disease status, especially at intermediate values of the PRS [21], possibly because translating population-level data to individual status is problematic and risks falling into the ecological fallacy. Many of the variants and their effect sizes are derived from a limited number of ancestral groups, as most research is done in European populations [58], potentially leading to a lack of PRS transferablity across populations [35]. Nonetheless, when a trait is multigenic or polygenic, a polygenic risk score is becoming an often used risk estimator. The role of the PRS to estimate prevalances among populations has not been explored as much as for individual risk, but it may point to key factors that are common. One study on height in admixed European/African populations found that the prediction ability of a polygenic risk score (PRS) for height was a function of the amount of European ancestry, supporting the idea that population-specific effect sizes and allele frequencies are important to its utility [2]. One recent example is PRS-CSx that uses GWAS summary statistics from multiple populations to improve the cross-ethnic transferability of PRSs [51]. Another study by Evans et al. used individual PRSs to estimate population-level disease prevalence [11], but the idea of using PRSs at a population level remains novel. In addition, Boyle et al. [3] have suggested that for traits affected by many loci, such as height, even “a small shift in average allele frequencies could generate a large shift in average height, e.g., a 0.5% genome-wide increase in the frequency of ‘‘tall’’ alleles would generate a 15 cm shift in average height” [3].

A polygenic risk score additively (i.e., without any interactions) incorporates some, or all, of the known risk associated loci for a phenotype, often identified by GWAS. On an individual level, polygenic risk scores (PRS) have been used to predict phenotypic status. This has been done with varying success. For some individuals in some diseases, a PRS can be a better predictor than monogenic markers in simple genetic diseases at the extremes of the PRS value distribution [27]. For others, some common diseases are very difficult to model using a PRS, probably due to complex genetic architecture and nongenetic factors [61]. Some studies only see modest improvement in risk prediction over only clinical risk factors, indicating that more information is needed to understand genetic architecture of diseases to translate PRS usage into clinical settings [49, 63], 65. PRSs can be calculated with or without estimated effects sizes. Weighted PRSs include the average effects of these alleles based on extant studies and sometimes the population allele frequency [6, 28]. Weighted PRSs are sometimes used to confirm associations in GWAS [67]. However, as effect sizes can vary among populations, this approach may present serious limitations [69].

PRSs have already been implemented for individual risk of many different phenotypes. For some diseases, a PRS can be used as an early indicator before other predictors present, allowing for preventative intervention. For example, individual risk of coronary artery disease can be stratified using an effect size weighted PRS [50]. Jia et al. [24], using GWAS cancer hits with data from the UK Biobank, found that PRSs could predict an individual’s elevated risk of several types of cancer, including prostate, breast, pancreas, colorectal, ovarian, lung, bladder, and kidney. However, Jia et al. [24] only included individuals of European descent and a relatively short follow-up time [24]. PRSs have also been shown to work well in the stratification and subtyping of breast cancer [37].

There is some variability in the conceptualization of PRSs and some confusion in the language used to define them. In addition, there have been numerous models and statistical constructs used to generate PRSs. For example, three general conceptualizations have been described [62]. The first general approach described uses only statistically significant GWAS SNPs, i.e., those where *p* < 5 × 10^–8^. This was deemed restricted-to-significant polygenic scores (rsPSs). In other studies, the PRS refers to scores that also incorporate non-significant SNPs with *p*-values ≥ 5 × 10^–8^. These were referred to as global extended polygenic scores (gePSs). A final score was proposed, process-specific polygenic scores (pPSs) that built the metric using variants grouped according to common biological processes or pathways [63]. With respect to different statistical constructs for use in individuals, there have been several previous PRS building methods. *P*-Value thresholding (p_T_) uses a *p*-value significance threshold for SNP selection [23]. LDpred2 derives polygenic scores based on summary statistics and a correlation matrix between genetic variants, i.e., those that are in LD [45]. A LASSO/Elastic Net method, lassosum, estimates variable selection based on a linear regression from GWAS summary statistics [34]. However, at present there is still not a single PRS construction method that has shown universal utility, although reporting standards have now been proposed that may move the field to more common standards [66].

For traits with high heritability and risk alleles common among populations, we hypothesize that a PRS weighted by risk allele frequencies, as a population average measurement, will associate with relative disease prevalence among populations as has been indicated previously [9]. While this is certainly true for Mendelian traits, we are proposing to ascertain the extent to which it is true for increasingly complex diseases. In theory, we should be able to predict relative ranking of disease prevalence among populations for simple traits, for which our understanding is relatively comprehensive with respect to the number and effect of risk loci. This will, presumably, allow us to predict population prevalence based on the correlation between prevalence and risk allele frequency using a risk score and assuming locus additivity. We hypothesize that risk allele frequencies as compiled into a PRS are proportional to population prevalence and the change in prevalence based on specific variants is proportional to their importance in disease presentation, i.e., effect size, among populations. In this paper, we assessed the ability of PRSs, in traits of varying presumed genetic complexity, to explain population differences in prevalence and to evaluate whether the components in a PRS act additively in their contribution to disease prevalence. We also examined whether SNPs have universal effects by adjusting the number in each PRS.

## Materials and methods

### Phenotypes

As proof of principle, we explored four phenotypes of differing presumed genetic complexity: lactase persistence, melanoma, multiple sclerosis, and height. While these traits are not exhaustive of the entire spectrum of genetic architecture, they appear to differ from each other in terms of genetic complexity and, should be broadly representative. As lactase persistence is monogenic, albeit with allelic heterogeneity, it is a genetically simple trait. Melanoma is dependent on both environment and a small number of known loci and is therefore likely oligogenic. Multiple sclerosis is a presumably moderately complex polygenic trait, with hundreds of associated alleles and several environmental factors. Height is a highly complex and heritable phenotype with thousands of associating alleles, making it essentially omnigenic.

### Lactase persistence

Lactase persistence into adulthood is a monogenic autosomal dominant trait caused by one or more of several mutations affecting the expression of lactase (*LCT*), the gene responsible for the encoding of lactase. Lactase persistence is reasonably well understood genetically in some, but not all, populations. Lactase is the enzyme that our bodies produce to help breakdown lactose, the sugar found in milk. The production of lactase usually decreases after weaning, in some cases leading to an intolerance of lactose. Lactase persistence shows strong evidence of selection, although why and when is a matter of debate [15, 44, 53, 54]. It is, however, believed to be associated with the advent of dairy farming. Individuals who are lactose intolerant can often consume a moderate amount of dairy, especially if processed into foods such as cheese and yogurt.

In Europe, two alleles upstream of the *LCT* gene, − 13,910*T (rs4988235) and − 22,018*G (rs182549), have been identified as conferring lactase persistence. These two SNPs are in strong LD in Europeans (*r*^2^ = 1.0 in all European populations from 1000 Genomes, except TSI where *r*^2^ = 0.95). In populations outside of Europe, other alleles have been associated with lactase persistence, where it exists [25, 31, 47, 60]. A total of 11 SNPs have been associated with lactase persistence (Additional file 15: Table S1). The prevalence of lactase persistence varies among populations around the world. For example, 92% of people in Great Britain (GBR) are lactase persistent, whereas, in Vietnam (KHV), the prevalence is only 2% (Table 1). We tested the expected relative frequency of lactase persistence based on a PRS, including all of the variants known to date, to see if we could predict relative prevalences, especially in populations that appear to carry the less penetrant alleles.

**Table 1:** Relative trait distribution among populations

Super population	Population	Lactase persistence	Melanoma	Multiple sclerosis	Male height (cm)	Female height (cm)
AFR	ACB	–	4.2 × 10^–5^ [12]	1.36 × 10^–4^ [8, 64]	175.9 [7]	165.3 [7]
	ASW	0.25 [1]	2.9 × 10^–5^ [18]	–	175.5 [13]	162.6 [13]
	ESN	0.13 [59]	5.7 × 10^–6^ [12]	3.71 × 10^–5^ [8, 64]	165.9 [7]	156.3 [7]
	GWD	0.43	0 [12]	3.35 × 10^–5^ [8, 64]	165.4 [7]	160.9 [7]
	LWK	0.61 [59]	1.4 × 10^–5^ [12]	3.30 × 10^–5^ [8, 64]	169.6 [7]	158.2 [7]
	MSL	0.52	5.6 × 10^–6^ [12]	2.89 × 10^–5^ [8, 64]	164.4 [7]	156.6 [7]
	YRI	0.13 [59]	5.7 × 10^–6^ [12]	3.71 × 10^–5^ [8, 64]	165.9 [7]	156.3 [7]
AMR	CLM	0.2 [59]	1.08 × 10^–4^ [12]	5.53 × 10^–5^ [8, 64]	169.5 [7]	156.95 [7]
	MXL	0.52 [59]	6.9 × 10^–5^ [12]	1.08 × 10^–4^ [8, 64]	169.0 [7]	156.9 [7]
	Px10L	0.06 [1]	8.3 × 10^–5^ [12]	6.98 × 10^–5^ [8, 64]	165.2 [7]	152.9 [7]
	PUR	–	1.11 × 10^–4^ [12]	1.9 × 10^–4^ [8, 64]	172.1 [7]	159.2 [7]
EAS	CDX	0.15 [59]	1.5 × 10^–5^ [12]	7.30 × 10^–5^ [8, 64]	171.8 [7]	159.7 [7]
	CHB	0.15 [59]	1.5 × 10^–5^ [12]	7.30 × 10^–5^ [8, 64]	171.8 [7]	159.7 [7]
	CHS	0.15 [59]	1.5 × 10^–5^ [12]	7.30 × 10^–5^ [8, 64]	171.8 [7]	159.7 [7]
	JPT	0.27 [59]	5.0 × 10^–5^ [12]	3.62 × 10^–4^ [8, 64]	170.8 [7]	158.3 [7]
	KHV	0.02 [59]	4.3 × 10^–6^ [12]	4.41 × 10^–5^ [8, 64]	164.5 [7]	153.6 [7]
EUR	CEU	0.87	1.3 × 10^–3^ [18]	–	177.4 [13]	163.3 [13]
	FIN	0.81[59]	1.04 × 10^–3^ [12]	1.49 × 10^–3^ [8, 64]	179.6 [7]	165.9 [7]
	GBR	0.92 [59]	9.39 × 10^–4^ [12]	1.61 × 10^–3^ [8, 64]	177.5 [7]	164.4 [7]
	IBS	0.71 [59]	3.92 × 10^–4^ [12]	9.41 × 10^–4^ [8, 64]	176.6 [7]	163.4 [7]
	TSI	0.28 [59]	7.12 × 10^–4^ [12]	1.19 × 10^–3^ [8, 64]	177.8 [7]	164.6 [7]
SAS	BEB	0.175	6.5 × 10^–6^ [12]	1.42 × 10^–4^ [8, 64]	163.8 [7]	150.8 [7]
	GIH	0.39 [59]	5.4 × 10^–6^ [12]	1.54 × 10^–4^ [8, 64]	165.0 [7]	152.6 [7]
	ITU	0.39 [59]	5.4 × 10^–6^ [12]	1.54 × 10^–4^ [8, 64]	165.0 [7]	152.6 [7]
	PJL	0.42 [59]	4.6 × 10^–6^ [12]	1.46 × 10^–04^ [8, 64]	167.0 [7]	153.8 [7]
	STU	0.27 [59]	1.4 × 10^–5^ [12]	3.35 × 10^–05^ [8, 64]	165.7 [7]	154.6 [7]

### Melanoma

A moderately complex oligogenic disease with 39 associated GWAS SNPs (Additional file 15: Table S2), melanoma is a skin cancer that is both heritable and dependent, to an extent, on environmental factors, especially ultraviolet (UV) exposure. Although considered rare, melanoma is responsible for most skin cancer deaths and the incidence is increasing, due partially to improved diagnosis [5]. Most cases of melanoma are caused by somatic mutations from exposure to UV light, although the above noted germline variants have been identified as conferring risk. Visualization with LocusZoom plot showed that SNPs associated with melanoma are distributed across the genome (Additional file 1: Figure S1) [46].

There is significant variation in melanoma prevalence globally, with the lowest rate in Vietnam and highest in Finland (FIN) (Table 1). As melanin is protective, melanoma is higher in prevalence in populations of lighter skin color. However, non-European populations have a higher risk of mortality, possibly because melanoma is harder to detect in darker skin, and detection and treatment are late in the course of the disease [10]. There is some indication, also, that skin color modifies the genetic architecture of melanoma [20].

The heritability of melanoma ranges from 19 to 58% [33, 39], 55. However, while known melanoma predisposing genes range in penetrance and frequency, known genes only explain ~ 50% of the heritability in families, indicating missing heritability and uncertain genetic architecture [48].

### Multiple sclerosis

Multiple sclerosis (MS) is an autoimmune neurologic disorder affecting the central nervous system. It is a relatively complex phenotype, dependent on both environmental exposures and genetics. Environmental factors include past Epstein-Barr virus infection, vitamin D insufficiency [42, 43], and cigarette smoking. MS also has a "latitude-gradient effect," i.e., the prevalence of MS is greater at higher latitudes, but there are some exceptions within Italy and Scandinavia [57]. World-wide prevalence of MS is shown in Table 1, except that we were unable to find representative, recent data on the European American (CEU) and African American (ASW) populations, as current prevalence data derive from military data in mostly males.

372 SNPs have been identified by GWAS as associating with MS (Additional file 15: Table S3). Visualization with LocusZoom showed that the SNPs are widely distributed across the genome (Additional file 2: Figure S2) [46]. Estimates of both prevalence and heritability vary among studies. MS is more common in women (70–75% of cases) [52] and people of European descent [38]. Studies vary on the heritability of MS; one done in Australia, multiple European countries, and US states shows moderate heritability (~ 20%) [22], although a Swedish study shows a much higher heritability of 64% (36–76%) [68].

### Height

As a truly polygenetic trait, human height is both complex and highly heritable [29, 30]. In addition to the 4388 variants currently found to associate with this phenotype by GWAS, height is also dependent on environmental factors, including diet (Additional file 15: Table S4) [70]. Visualization LocusZoom showed the SNPs are widely distributed across the genome (Additional file 3: Figure S3) [46]. There are also differences in average height between men and women and between global populations. The average height for men ranges from 163.8 cm in Bangladesh to 179.6 cm in Finland. For women, average height ranges from 150.8 to 165.9, also in Bangladesh and Finland, respectively (Table 1). Height is less heritable in women than men (0.68 to 0.84 vs. 0.87 to 0.93, respectively) [56]. Male and female population average heights are highly, but not completely, correlated (*r*^2^ = 0.84), potentially leading to some differences in the genetic models between sexes.

### Allele and prevalence data collection

Associated alleles for each phenotype were identified by a literature search and accessing the alleles that have been identified by GWAS from the GWAS Catalog at *p* < 1 × 10^−5^. We chose to use this as the threshold for significance in our initial analyses, but report difference by *p*-value threshold as well. Prevalence data for each phenotype in each population came, similarly, from literature searches and from databases devoted to specific traits (cancer, height). For lactase persistence, we found the associated SNPs, through literature search for lactose intolerance, and it was necessary to subtract the proportion of lactose intolerance in a population from 1. For melanoma, we accessed the associated SNPs in the GWAS Catalog under the trait melanoma (GWAS Catalog identifier: EFO_0000756). Multiple sclerosis SNPs were obtained from the GWAS Catalog under the trait multiple sclerosis (EFO_0003885). The GWAS Catalog trait body height (EFO_0004339) was used for the height SNPs. An attempt was made to keep the sources as similar as possible for each population (Table 1).

### 1000 Genomes

To assess the role of PRSs in predicting population phenotype distributions, we chose to use only the populations included in The International Genome Sample Resource (IGSR) from the 1000 Genomes Project (Additional file 15: Table S5) as our study populations. Each ancestral population in the IGSR belongs to a larger super-population defined as: East Asian (EAS), South Asian (SAS), European (EUR), African (AFR) and Ad Mixed American (AMR). The frequencies of known risk alleles defined in the GWAS Catalog and literature were extracted from the 1000 Genomes data using the Ensembl REST API.

### Polygenic risk scores

Under the assumption that the genetic architecture of a phenotype is additive, we used a PRS to account for the genetic risk in each of our study populations, based on the frequency of the disease-causing alleles to estimate the relative presence of the phenotype in that population. As previously mentioned, in individuals this is done by simply summing the number of risk alleles that an individual possesses, usually GWAS hits, for the specific phenotype. Another approach is to weight each allele in the score by the effect size and/or the allele frequency. However, for a population-specific PRS (psPRS), effect sizes may not be transferable [35, 36, 58], and as long as the direction of effect is the same, the role that any variant plays in prevalence should be proportional to the frequency of the risk allele in that population. We have structured psPRSs without effect size weighting, as there is often little to no information on effect sizes/OR of the risk alleles in different populations. Therefore, we calculated our psPRSs only by the population allele frequencies. In addition, many of the associating SNPs do not have reported effect sizes in the data sources available. Our expression for the psPRSs is simply the sum of the frequencies for the risk alleles in each population. For a population in the 1000 Genomes database, psPRS is the PRS for that population and *p*_*i*_ is the allele frequency of SNP_*i*_:

$${\mathrm{psPRS}}=\sum_{k=1}^{i}{p}_{k}$$

We then performed a linear regression between the sum of the risk allele frequencies in a population and the prevalence of the phenotype to establish the relationship between the population-specific psPRS and the population prevalence of that phenotype (Additional file 15: Table S6).

### Maximization of the coefficient of determination sensitivity analysis

We performed a sensitivity analysis, filtering SNPs based on maximizing the coefficient of determination (*r*^2^), or the square of the coefficient of correlation (*r*). This analysis used a process of elimination of SNPs that reduced the predictiveness of each psPRS. This was done by assessing the effect of removing SNPs from the psPRS and ordering each SNP by the *r*^2^ value calculated for the linear regression between the population psPRS without that SNP and the population prevalence. The SNPs that resulted in the model where the *r*^2^ was the largest were retained, while the SNPs that reduced the predictability were discarded. We then recalculated the *r*^2^ values for the model with only the remaining SNPs (Additional file 15: Tables S1–S4, S7 and Additional files 9–13: Figures S9-S13). We repeated this process until the *r*^2^ value reached a maximum and only the most predictive SNPs remained. The code for our algorithm is in the supplement methods (Additional file 14).

This method empirically prioritizes the SNPs that best predict trait prevalence. Under the assumption of additivity, the model with the largest *r*^2^ was expected to include all truly associating SNPs with universal effects (Table 2). Our approach tested this implicitly.

**Table 2:** Sensitivity analysis

Phenotype	GWAS SNPs	1000 Genomes SNPs	Reduced SNPs
Lactase persistence	11	NA	4
Melanoma	39	37	16
Multiple sclerosis	372	368	131
Height male	4388	4209	547
Height female	4388	4209	188

## Results

### Lactase persistence

**Fig. 1:**
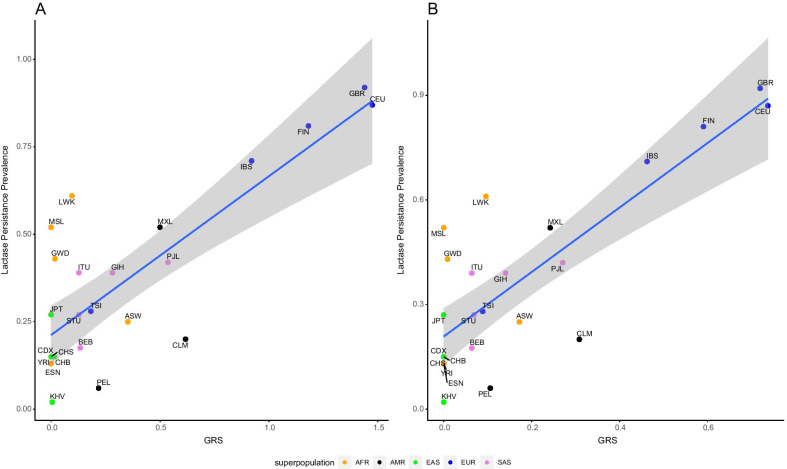
Correlation between lactase persistence and psGRS. The data points are colored according to the super populations: AFR (orange), AMR (black), EAS (green), EUR (blue) and SAS (purple). The scale of the x-axis is not the same for both plots due to differing psPRS ranges. **A** Full model (r^2^ = 0.65; *p*-value: 1.84 × 10^−06^). **B** After maximization (r^2^ = 0.67, *p*-value: 9.13 × 10^−07^). 1000 Genome populations are as follows: *CHB*—Han Chinese in Beijing, China; *JPT*- Japanese in Tokyo, Japan; *CHS* -Southern Han Chinese; *CDX*—Chinese Dai in Xishuangbanna, China; *KHV*—Kinh in Ho Chi Minh City, Vietnam; *CEU*—Utah Residents (CEPH) with Northern and Western European Ancestry; *TSI*—Toscani in Italy; *FIN*—Finnish in Finland; *GBR*—British in England and Scotland; *IBS*—Iberian Population in Spain; *YRI*—Yoruba in Ibadan, Nigeria; *LWK*—Luhya in Webuye, Kenya; *GWD*—Gambian in Western Divisions in the Gambia; *MSL*—Mende in Sierra Leone; *ESN*—Esan in Nigeria; *ASW*—Americans of African Ancestry in SW USA; *ACB*—African Caribbean in Barbados; *MXL*—Mexican Ancestry from Los Angeles USA; *PUR*—Puerto Ricans from Puerto Rico; *CLM—C*olombians from Medellin, Colombia; *PEL—*Peruvians from Lima, Peru; *GIH—*Gujarati Indian from Houston, Texas; *PJL*—Punjabi from Lahore, Pakistan; *BEB*—Bengali from Bangladesh; *STU* -Sri Lankan Tamil from the UK; *ITU*—Indian Telugu from the UK

We identified 11 SNPs associated with lactase persistence in the literature (Additional file 15: Table S1). We used these SNPs to build our LP PRS for each population, using allele frequencies from the 1000 Genomes Project. We found a strong relationship between the PRS and the population prevalence of lactase persistence with a *r*^2^ value of 0.65 (Fig. 1A, *p*-value: 1.84 × 10^−06^).

The relationship was especially strong among European populations, but less so for South Asian and Amerindian populations. However, in East Asian and African populations, the PRS failed to account for much, if any, of the relationship between the known lactase persistence alleles and the population prevalence (Additional file 4: Figure S4A). The sensitivity analysis (Additional file 9: Figure S9, Additional file 15: Table S1) based on *r*^2^ maximization showed that keeping only four specific SNPs (Additional file 15: Table S8) maximized the predictability (*r*^2^ = 0.67, *p*-value: 9.13 × 10^−07^) and, although the *r*^2^ did not increase by much (0.65–0.67), the slope of the linear regression changed from 0.45 to 0.92 (Fig. 1B). The position of the populations that had high European allele content changed quite a bit, as one of the alleles that were filtered out of the PRS was one of two alleles originally identified in Europeans. This is not a surprise, because the European alleles are in strong LD (in the European populations), and, of the alleles tested, these are the only ones in LD in Europe.

Within super populations, the relationships varied considerably. In the African subpopulations, the trend of the linear regression was slightly negative before maximization but slightly positive after (Additional file 4: Figure S4B). The admixed African American population (ASW) has the highest PRS, but relatively low prevalence of LP (25%). This is due to the presence of the European alleles in the ASW population that are not present in West Africa. In the East Asian populations (EAS), the trend is also negative, but after the maximization of *r*^2^, there were no SNPs retained that existed in the EAS populations. In the European populations (EUR), the trend was positive and stayed positive after maximization, as expected given the relative frequency of the European derived LP alleles, ranging from 0.09 in the TSI to 0.73 in the CEU. In the South Asian populations (SAS), the trend was again positive and stayed so after maximization.

### Melanoma

**Fig. 2:**
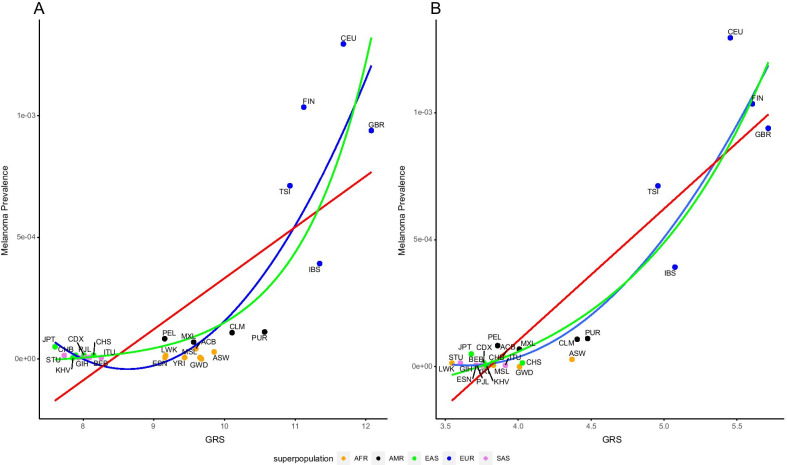
Correlation between melanoma and psGRS with regression lines for linear (red), polynomial (blue) and exponential (green) relationships. The data points are colored according to the super populations: AFR (orange), AMR (black), EAS (green), EUR (blue) and SAS (purple). The scale of the x-axis is not the same for both plots due to differing psPRS ranges. **A** Full model with three regressions: polynomial (*r*^2^ = 0.78, *p*-value: 2.19 × 10^−07^), exponential (*r*^2^ = 0.66, *p*-value 2.7 × 10^−06^) and linear (*r*^2^ = 0.59; *p*-value: 1.71 × 10^−05^). **B** After maximization: linear regression (*r*^2^ = 0.88, *p*-value: 2.81 × 10^−11^), polynomial (*r*^2^ = 0.94, *p*-value: 7.36 × 10^−13^) and exponential (*r*^2^ = 0.77, *p*-value: 3.39 × 10^−08^). Populations as defined in Fig. 1

Thirty seven of the 39 GWAS SNPs were also in the 1000 Genomes Project (Additional file 15: Table S2). The relationship of the melanoma psPRS with these 37 associating SNPs to the population prevalence appears to be nonlinear (Fig. 2A). We applied three different types of regression: linear, polynomial and exponential. The one that explained the relationship the best was the second-order polynomial regression (*r*^2^ = 0.78, *p*-value: 2.19 × 10^−07^); the exponential model was next best (*r*^2^ = 0.66, *p*-value 2.7 × 10^−06^) and the linear the worst (*r*^2^ = 0.59; *p*-value: 1.71 × 10^−05^), although all were significant. The overall relationship of the psPRSs and the population prevalences reflects the fact that the highest prevalence and psPRSs are in European populations. East Asian populations had the lowest PRSs and prevalence. South Asian populations clustered with some Amerindian populations with low to medium PRSs. African populations had medium PRSs, but low melanoma prevalence.

With the 16 SNPs that remained after the maximization analysis (Additional file 15: Table S9, Additional file 10: Figure S10), the relationship between the melanoma population PRS and the population prevalence appeared to remain nonlinear, similar to the original model, but with an improved explanation of variance and significance (linear regression: *r*^2^ = 0.88, *p*-value: 2.81 × 10^−11^) (Fig. 2B). We also explored both polynomial (*r*^2^ = 0.94, *p*-value: 7.36 × 10^−13^) and exponential relationships (*r*^2^ = 0.77, *p*-value: 3.39 × 10^−08^). These models all performed better than the full psPRS model.

When we separated populations according to their super populations, we observed that, apart from the Asian populations, the correlations were positive, but of varying strength (Additional file 5: Figure S5A). However, none of the relationships were significant, perhaps due to the relatively small sample size. These results indicate that the significant correlation is driven by the relationship among the continental populations that are not identical to each other. After maximization, the positive and negative trends were as described above, with the Asian populations staying negative and the EUR, AFR, and AMR remaining positive (Additional file 5: Figure S5B). The correlations did not improve substantially within super populations and remained non-significant using the reduced number of SNPs.

### Multiple sclerosis

**Fig. 3:**
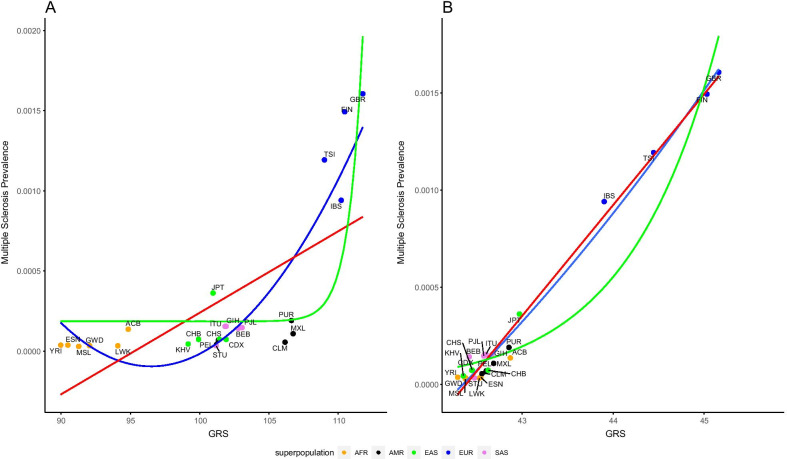
Correlation between multiple sclerosis and psGRS with linear (red), polynomial (blue) and exponential (green) regressions lines. The data points are colored according to the super populations: AFR (orange), AMR (black), EAS (green), EUR (blue) and SAS (purple). The scale of the x-axis is not the same for both plots due to differing psPRS ranges. **A** Full model with three regression types: polynomial (*r*^2^ = 0.80, *p*-value: 3.94 × 10^–08^), linear, (*r*^2^ = 0.47, *p*-value: 2.12 × 10^–04^) and exponential (*r*^2^ = 0.64, *p*-value: 2.59 × 10^–06^). **B** Linear regression after maximization (*r*^2^ = 0.98, *p*-value: 9.9 × 10^–11^). Populations as defined in Fig. 1

For the full psPRS-prevalence multiple sclerosis model, we used 368 SNPs associated with MS that were in both the GWAS Catalog and the 1000 Genomes Project (Additional file 15: Table S3). The resulting relationship appears to be nonlinear (Fig. 3A). We explored three different models for the regression: linear, polynomial and exponential. As with melanoma, the model that explained the largest proportion of the variance was the second order polynomial (*r*^2^ = 0.80, *p*-value: 3.94 × 10^−08^). The worst was the linear model (*r*^2^ = 0.47, *p*-value: 2.12 × 10^−04^), while the exponential model was intermediate (*r*^2^ = 0.64, *p*-value: 2.59 × 10^−06^).

After the *r*^2^ maximization sensitivity analysis (Additional file 15: Table S10, Additional file 11: Figure S11), the filtered PRS model included 131 SNPs and appears to be best modeled linearly (Fig. 3B, *r*^2^ = 0.98, *p*-value: 9.9 × 10^−11^). The linearity remains, even when the European populations are removed. Within the super populations, the prevalences and psPRSs become more highly correlated and the relationships, apart from the South Asian populations, are significant (Additional file 6: Figure S6A).

The super populations clustered, with the European populations having the highest prevalence and psPRSs. The African populations had the lowest PRSs and prevalences, with the east and south Asian mixed with the admixed Amerindian with medium prevalences and PRSs. When the super populations were examined individually, the linear correlations were all positive, with strengths ranging from EAS (*r*^2^ = 0.0459) to AFR (*r*^2^ = 0.4336). However, again, none of these relationships were significant (Additional file 6: Figure S6B).

### Height

**Fig. 4:**
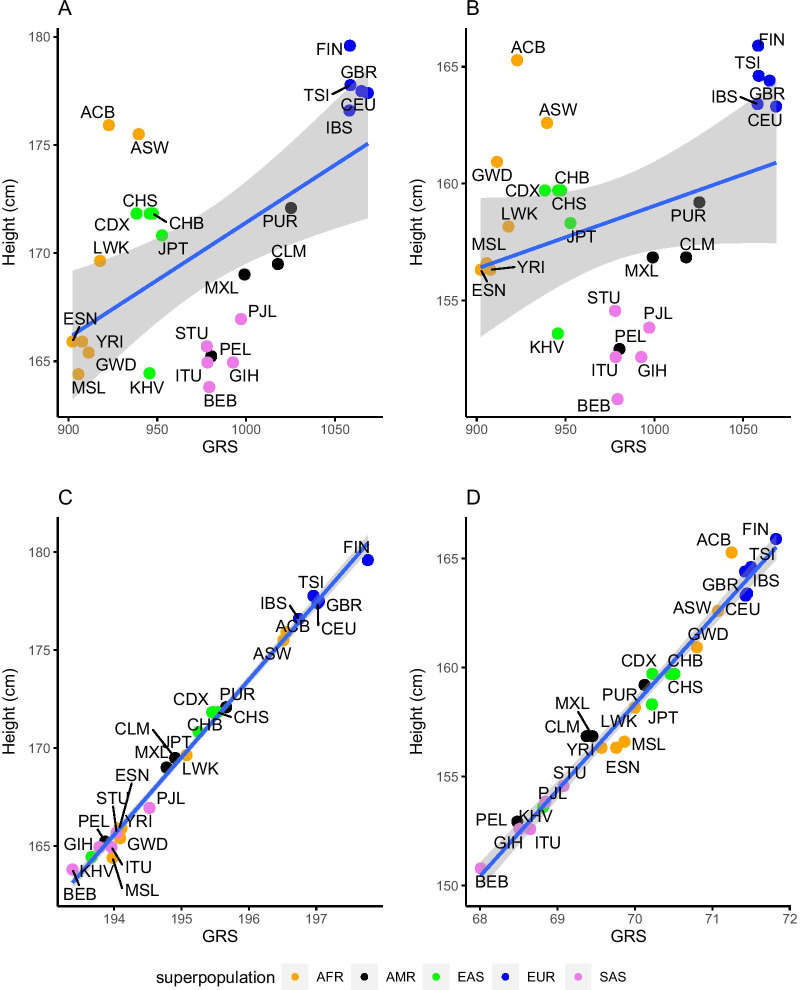
Correlation between height (cm) and psGRS. The data points are colored according to the super populations: AFR (orange), AMR (black), EAS (green), EUR (blue) and SAS (purple). The scale of the x-axis is not the same for both plots due to differing psPRS ranges. **A** Full model male linear regression (*r*^2^ = 0.32, *p*-value: 2.55 × 10^−03^). **B** Full model female linear regression (*r*^2^ = 0.11, *p*-value: 0.0992). **C** Male linear regression after maximization (*r*^2^ = 0.99, *p*-value: < 2 × 10^−16^). **D** Female linear regression after maximization (*r*^2^.98, *p*-value: < 2 × 10^−16^). Populations as defined in Fig. 1

Because height has quite different ranges for men (~ 164 cm to ~ 180 cm) and women (~ 151 cm to ~ 166 cm) (Table 1), we examined the relationship between population average height and population PRS in each sex separately. The full psPRS average height model included 4208 SNPs from the GWAS Catalog and the 1000 Genomes Project for both men and women (Additional file 15: Table S4). The relationships for both male and female between the population PRSs and the population average height (cm) appear to be linear (Fig. 4A and B). However, the regressions for men and women are different, with noticeable differences in the slopes of the regression lines, the correlations, and the significance of the relationships (male: *r*^2^ = 0.32, *p*-value: 2.55 × 10^−03^; female: *r*^2^ = 0.11, *p*-value: 0.0992).

The populations generally clustered by super populations, with European populations being both the tallest and having the largest psPRSs for both men and women. The south Asian and Amerindian were the shortest groups, but with medium PRSs. African and east Asian populations had medium to tall height, but the lowest PRSs.

Within the African super population, the relationship between average height and population PRSs was positive in both males and females. Both South Asian and Amerindian populations had positive relationships as well. However, surprisingly, the European and east Asian populations had negative relationships (Additional file 7: Figures S7A and Additional file 8: Figure S8A).

The sensitivity analysis reduced the number of SNPs for the male model to 548 and for the female model to 188 (Fig. 4C, D, Additional file 12: Figure S12, Additional file 13: Figure S13, Additional file 15: Tables S4, S7, S11–S12). The reduced male and female linear models changed substantially (male: slope from 0.06 to 3.92; female: slope from 0.03 to 3.86) due the narrower range of the independent variable. The correlation strengthened for both male and female (male: *r*^2^ = 0.99; female: 0.98), and in males the relationship became more significant and became significant in females (male and female: *p*-value: < 2 × 10^−16^).

After the maximization filtering, the positions of the populations shifted significantly. The South Asians had lower PRSs to match their lower average height. Europeans still had the highest psPRSs and the African and east Asian populations were mixed (Fig. 4C, D). Within the super populations, the relationships all became positive for both men and women (Additional files 7 and 8: Figures S7B and Additional file 5: Figure S8B).

### Effect of *p*-value thresholds for SNP selections

As we used only a moderately stringent threshold for the SNPs from the GWAS Catalog, we wished to know if the maximization analysis selected SNPs that were more likely to be statistically significant, i.e., with *p*-values of genome-wide significance. We found, using the Fisher’s exact test, that there was no significant enrichment of GWAS SNPs with a *p*-value less than 5 × 10^−8^, except for height in women (Table 3).

**Table 3:** *P*-value enrichment analysis

Phenotype	Melanoma		Multiple Sclerosis		Male Height		Female Height	
Threshold	Unfiltered	Filtered	Unfiltered	Filtered	Unfiltered	Filtered	Unfiltered	Filtered
GWAS significant^1^	27	12	199	71	3552	478	3552	188
GWAS not significant^2^	10	4	169	60	656	69	656	0
*p*-value		1		1		0.07644		6.73E−14

^1^*P* ≤ 5 × 10^–8^

^2^ 1 × 10^–5^ > *P* > 5 × 10^–8^

### Association between allele frequency and SNP pruning

To assess whether the pruning method preferentially selects SNPs with a different allele frequency than the full model, we compared the average frequency for each allele across all of the 1000 Genomes sub populations. We then compared the mean frequency of the full SNP set for each phenotype to that of the pruned sets. For LP and melanoma, the differences between the full set frequency means and those of the pruned set were not significant. However, for MS and height the differences in means between the full and pruned sets were statistically significant with the pruned alleles being on average more common (MS allele frequency mean: full = 0.2718688, pruned = 0.3284336, *p*-value = 1.72 × 10^−6^; height allele frequency mean: full = 0.2321003, filtered male = 0.3567876, *p*-value < 2.2 × 10^−16^, filtered female = 0.3705134, *p*-value < 2.2 × 10^−16^). We also compared the means of the male and female pruned sets and found that there was not a statistically significant difference.

### Physical location of psPRS SNPs

It is possible that LD between SNPs extracted from the GWAS Catalog for inclusion in the psPRS could affect results with SNPs in LD having disproportionate influence. However, as we are using 26 distinct populations there is unlikely to be a common pattern of LD. To visualize the possible impact of LD, we plotted the full SNP sets using LocusZoom [46]. The pre-pruned SNP plots for melanoma, MS, and height were broadly distributed across the genome. The average physical distance between SNPs on each chromosome in psPRS is usually high. In the reduced models, the average distance between SNPs, goes up, as is expected in most cases (melanoma: full = 28,578,964 bp, pruned = 26,122,334 bp; MS: full = 6,815,595 bp, pruned = 15,131,023 bp; height: full: 661,350 bp, male pruned: 4,675,948 bp, female pruned: 11,859,376 bp).

SNPs that were very close, and most likely in LD, were also determined. For height, approximately 27% of the SNPs (1,128) were within 30.8 KB of each in the full dataset and in the reduced datasets 9.6% to 15.0% (82 in males and 18 in female) were within this distance. For melanoma, 5 of 37 SNPs were within 30.8 kb of each other, as were 93 of 268 for multiple sclerosis in the full data sets. The pruned SNPs for these two traits that were within this distance were 0 of 16 and 18 of 131, respectively. In all cases, the pruning reduced the proportion of nearby SNPs, probably preferentially removing SNPs in LD.

## Discussion

Overall, our psPRS method estimated population prevalence quite well. This indicates that the population PRS is a reasonably good predictor of disease presence in a population. For lactase persistence, we found that the psPRSs and the prevalence were strongly correlated, even before SNP filtering. For melanoma and MS, we also found strong, albeit nonlinear, correlations. However, for height, the correlations, while linear, were weaker. As expected, the complexity of the phenotype did affect the ability of the full PRS model to predict the population prevalence, sometimes being far from what would have been expected and being nonlinear, i.e., melanoma and MS. Also, the pruned models improved the explained variance over the complete psPRSs, sometimes substantially, and the relationships achieved, or approached, linearity when the complete models did not. Although this can be viewed as “cherry picking,” the pruning does reveal that not all detected SNPs have similar effects across populations and that some may reflect effects that are universal as opposed to population specific. Our results show that the European populations often skew the overall full model and that, except for height, fit the PRS predictions best. This is not surprising as most of the SNPs were discovered in populations of European descent (Table 4) [58]. We also repeatedly observed that there were not as many significant correlations within the super populations, but there were between super populations, which may reflect the paucity of data within them. The 1000 Genomes superpopulations may not be representative of the best groupings of the subpopulations. For example, African populations are very diverse and, while the 1000 Genomes data are based on ethnic groups, the phenotypic prevalence data are based on country data. Geographical location and ethnicity are therefore often not equivalent. This could cause issues with the psPRS method, due to mis-assignment of phenotypes. However, the psPRSs still explain most of the global patterns of variation. Generally, the model of LP followed what was expected, as it is a monogenic disease. The global relationship appears to be driven mostly by the “European” alleles. For LP in the African populations, the disparity between the observed prevalence in some populations and our psPRS model shows that our ability to predict prevalence is likely impacted by unidentified associating alleles or other mechanisms by which lactose is digested, perhaps acquired gut microbiome activity [16]. This is supported by the negative and weak relationship in the full data set, although likely impacted by the admixed ASW population, where the European alleles exist but do not seem to confer lactase persistence to the extent that the psPRS would predict. Another possible reason for the psPRS not predicting prevalence in Africa well is that there may be context-dependent effects. For example, it has been found that the 13,915*G DNA polymorphism, associated with lactase persistence in Africa, interacts with *Oct-1* [40].

**Table 4:** Percentage of SNPs discovered in European populations

Trait	European	Non-European	Total	% European
LP	2	9	11	18.18
Height	3422	786	4208	81.32
MS	366	2	368	99.46
Melanoma	37	0	37	100

Given the known impact of environmental exposure on the development of melanoma [10], the observed nonlinearity of the relationship between the population PRSs and the prevalence of the disease was not unexpected. It appears that part of the nonlinearity is due to the European population cluster, as there is a greater amount of information in European populations, and this may to some extent, be skewing the phenotype-genotype relationship. That the nonlinearity continued after the filtering implies that the actual relationship between the psPRSs and the prevalence may be non-additive and that we are missing key factors, either genetic interactions, environmental interactions, or both. Because we did not consider environmental factors in this study, we were not able to differentiate between the two. It has, however, been shown that at least one SNP pair at the *TERF1* and the *AFAP1L2* loci does interact to affect risk of melanoma [4].

While the relationship observed with the full MS psPRS model was nonlinear, after filtering the SNPs, the resulting model was strongly linear. This might indicate that there is some genetic interaction in MS, especially given that the r^2^ improves as we drop SNPs from the psPRS model. Indeed, a *DDX39B* variant interacts with allelic variants in *IL7R* exon 6 to increase MS risk [14]. Interaction with environmental factors has also been shown. Specifically, latitude, EBV infection, smoking, and adolescent obesity interact with risk alleles at the *HLA* locus to increase risk of MS [41].

While the relationship of the full model psPRSs to population average height shows a relatively weak, though significant, positive correlation, the result of the maximization shows a very strongly correlated relationship. Although height is highly heritable, this was not expected, given the foreknowledge of the impact of environment on height, especially in women [56]. It may be that some of the variants left in the final model are correlated with environmental parameters due to past selection. Also, there may be epistasis in the genetic architecture of height. For example, genetic interaction was found between loci 6p21 and 2q21 to account some of the variation in height [32].

We infer from our results that the maximizing r^2^ sensitivity analysis is filtering out the SNPs that are not distributed as the population prevalence distribution of the phenotype in some, but maybe not all, populations. This is, in effect, similar to a previous method Evolutionary Triangulation [19], where we filtered SNPs based specifically on their *F*_st_ distribution relative to disease prevalence. Our results showing that pruning the SNPs in the model improves performance may be revealing heterogenous effect sizes that may present due to context dependent effects, such as epistasis or gene X environment interactions, spurious associations, or other population-specific effects. This may explain why a filtered model is superior in some cases to a model with all associating SNPs included. This approach is, in essence, removing noisy data. psPRSs provide some explanation of population differences but are less effective when all SNPs are included. This indicates that PRSs have value but must be refined to improve prediction. Although the underlying basis for improving prediction with a reduced number of SNPs and the nonlinear relationships between psPRSs and prevalence for some of the traits are unclear, we present above what we think are reasonable hypotheses worthy of further exploration. These findings may provide a novel means to explore the true genetic architecture of complex traits.

Our investigation as to whether GWAS significance was a useful threshold for inclusion indicated that *p*-value was not good at predicting which SNPs would end up in our pruned SNP set. As shown by our investigation of whether our model enriched for SNPs with a smaller *p*-value in the GWAS Catalog, we can conclude that GWAS p-value is not always the best indicator of the value of a SNP in the PRS model. This does justify, to some extent, our use of SNPs that were not genome-wide significant at 5 × 10^–08^ and indicates that some care should be used in determining the importance of SNPs in models based solely on significance of *p*-values, such as the *p*_T_ method. Our results cast uncertainty on how effective this method is in capturing relevant SNPs. Plotting the genomic positions of the SNPs for each phenotype showed that LD is unlikely to be an issue as the SNPs were distributed relatively evenly across the genome [46]. In addition, at least for LP, the pruning removed one SNP of the only pair in LD in Europeans.

For our analyses, we note that, of the SNPs we used to generate the psPRSs, the vast majority were discovered in populations of European descent, the exception being the SNPs for LP, where only two of the 11 SNPs were European determined. All SNPs associated with melanoma were discovered in European populations. For MS, two SNPs were discovered in non-European populations; 367 were from European descent populations. Out of 4208 SNPs associated with height, 3422 were discovered in European populations (Table 4). However, the psPRS is not always the largest in European populations. For example, lactase persistence in Tuscans is relatively low (28%) and the psPRS is correspondingly low. Nonetheless, the relationships within the European superpopulation tend to be among the best predicted based on the psPRS, probably because the data from those populations are the most complete. In fact, it is often true that, where the population prevalence is high in Europe, so is the psPRS and vice versa. It will be important to broaden the number and diversity of discovery populations so as to decrease this bias.

Understanding the relationship between allele frequency and disease prevalence will lead to further understanding of genetic influence, environmental pressure, and gene–environment interactions. The effects of genetic variation on public health present challenges for the exploration and management of these phenotypes worldwide, as most traits are primarily considered in the context of European descent. This blind spot, due partially to a lack of diversity in biomedical research, is not only detrimental to those populations that are understudied, but to the understanding of the underlying genetic basis, or genetic architecture, of the trait itself, thereby, possibly affecting understanding in all populations. Nonetheless, some of our results indicate that even SNPs discovered primarily in Europeans are useful, when included in a psPRS, for predicting trait variation, e.g., height.

A future extension of our method might be to use SNPs from the GWAS catalog to define loci of interest and then calculate the psPRS using all of the SNPs within that region so as to minimize the effects of population-specific LD with functional variants and then use our pruning method to further refine signals. This may identify SNPs that act only in specific contexts, such as variable genetic backgrounds (i.e., epistasis). Additionally, it may be beneficial to include SNPs that are less statistically significant than the ones that we did, especially considering our finding that the GWAS *p*-value did not differentiate pruned from unpruned SNPs well.

A comparison of the average allele frequency of the unpruned versus pruned sets of SNPs for each trait indicates that we are selecting more common SNPs through *r*^2^ maximization pruning at least for the more polygenic traits. That the more complex diseases show a significant difference in unpruned versus prunedallele frequency means is not unexpected because the SNP pool with less common variants may on average decrease statistical power to detect predictive alleles. Also, SNPs that are more common are most likely to be more broadly distributed.

Our results help to identify the populations in which we are missing the most information regarding genetic foundations of trait variation. This is underlined by some of our results where the population PRSs do not match the population prevalences, i.e., where the prevalence is high or medium and the psPRS is low, as in the cases of height and LP in African populations. That using a reduced number of SNPs improves the psPRS likely indicates a certain portion of missing heritability is due to more complex architecture, i.e., genetic interaction, possibly differing by population and that there are still undiscovered variants. However, our method helps to define the areas of the genetic landscape where our knowledge of genetic architecture is relatively complete and universal, and where it is not.

## Supplementary Information

**Additional file 1: Figure S1**. LocusZoom chromosomal location plot of the full melanoma SNP set.

**Additional file 2: Figure S2**. LocusZoom chromosomal location plot of the full multiple sclerosis SNP set.

**Additional file 3: Figure S3**. LocusZoom chromosomal location plot of the full height SNP set.

**Additional file 4: Figure S4**. Lactase persistence separated by super population. The data points are colored according to the super populations: AFR (orange), AMR (black), EAS (green), EUR (blue) and SAS (purple). A) Full model by super population: AFR (r^2^ = 0.0021, p-value: 0.9314), AMR (r^2^ = 0.2608, p-value: 0.659), EAS (r^2^ = 0.077, p-value: 0.6514), EUR (r^2^ = 0.9734, p-value: 0.00185) and SAS (r^2^ = 0.3847, p-value: 0.2643). B) Super populations after maximization: AFR (r^2^ = 0.0177, p-value: 0.8017), AMR (r^2^ = 0.2284, p-value: 0.683), EAS (no data), EUR (r^2^ = 0.9747, p-value: 0.00172) and SAS (r^2^ = 0.3914, p-value: 0.0580).

**Additional file 5: Figure S5**. Melanoma separated by super population. The data points are colored according to the super populations: AFR (orange), AMR (black), EAS (green), EUR (blue) and SAS (purple). A) Full model by super population: AFR (r^2^ = 0.1178, p-value: 0.5718), AMR (r^2^ = 0.6664, p-value: 0.1837), EAS (r^2^ = 0.4958, p-value: 0.1844), EUR (r^2^ = 0.0421, p-value: 0.7949) and SAS (r^2^ = 0.5914, p-value: 0.1285). B) Super populations after maximization: AFR (r^2^ = 0.1767, p-value: 0.481), AMR (r^2^ = 0.7766, p-value: 0.1187), EAS (r^2^ = 0.2399, p-value: 0.4022), EUR (r^2^ = 0.6268, p-value: 0.2083) and SAS (r^2^ = 0.4324, p-value: 0.2278).

**Additional file 6: Figure S6**. Multiple sclerosis separated by super population. The data points are colored according to the super populations: AFR (orange), AMR (black), EAS (green), EUR (blue) and SAS (purple). A) Full model super populations: AFR (r^2^ = 0.4336, p-value: 0.155), AMR (r^2^ = 0.1958, p-value: 0.5575), EAS (r^2^ = 0.0459, p-value: 0.7293), EUR (r^2^ = 0.3676, p-value: 0.3937) and SAS (r^2^ = 0.3775, p-value: 0.270). B) Super populations after maximization: AFR (r^2^ = 0.7781, p-value: 0.02003), AMR (r^2^ = 0.9821, p-value: 0.008995), EAS (r^2^ = 0.8775, p-value: 0.0189), EUR (r^2^ = 0.9988, p-value: 0.000617) and SAS (r^2^ = 0.2356, p-value: 0.407) .

**Additional file 7: Figure S7**. Male height separated by super population. The data points are colored according to the super populations: AFR (orange), AMR (black), EAS (green), EUR (blue) and SAS (purple). A) Super populations with full model: AFR (r^2^ = 0.7835, p-value: 0.00806), AMR (r^2^ = 0.8628, p-value: 0.0522), EAS (r^2^ = 0.1003, p-value: 0.9254), EUR (r^2^ = 0.578, p-value: 0.551) and SAS: r^2^ = 0.1162, p-value: 0.2812. B) super populations after performing maximization: AFR (r^2^ = 0.5534, p-value: 6.549 x 10-7), AMR (r^2^ = 0.8556, p-value: 0.001876), EAS (r^2^ = 0.0163, p-value: 2.052 x 10-5), EUR (r^2^ = 0.9158, p-value: 0.01064) and SAS (r^2^ = 0.0475, p-value: 0.002888).

**Additional file 8: Figure S8**. Female height separated by super population. The data points are colored according to the super populations: AFR (orange), AMR (black), EAS (green), EUR (blue) and SAS (purple). A) Super populations with full model: AFR (r^2^ = 0.5534, p-value: 0.05523), AMR (r^2^ = 0.8556, p-value: 0.07501), EAS (r^2^ = 0.0163, p-value: 0.8379), EUR (r^2^ = 0.2222, p-value: 0.4229) and SAS (r^2^ = 0.0475, p-value: 0.7246). B) Super populations after maximization: AFR (r^2^ = 0.9533, p-value: 0.0001627), AMR (r^2^ = 0.9917, p-value: 0.004174), EAS (r^2^ = 0.963, p-value: 0.003058), EUR (r^2^ = 0.754, p-value: 0.05619) and SAS (r^2^ = 0.9761, p-value: 0.001584).

**Additional file 9: Figure S9**. Lactase persistence maximization analysis r^2^ values.

**Additional file 10: Figure S10**. Melanoma maximization analysis r^2^ values.

**Additional file 11: Figure S11**. Multiple sclerosis maximization analysis r^2^ values.

**Additional file 12: Figure S12**. Male height maximization analysis r^2^ values.

**Additional file 13: Figure S13**. Female height maximization analysis r^2^ values.

**Additional file 14:** Script used for SNP pruning method.

**Additional file 15: Table S1**. Lactase persistence full data set. SNP rs number and minor allele are included, as well as the r2 values from the sensitivity analysis. The columns headed with the 1000 Genomes population codes are the allele frequencies for each SNP in those populations. The SNPs are listed in order of removal in the sensitivity analysis. **Table S2**. Melanoma full data set. SNP rs number and minor allele are included, as well as the r^2^ values from the sensitivity analysis. The columns headed with the 1000 Genomes population codes are the allele frequencies for each SNP in those populations. The SNPs are listed in order of removal in the sensitivity analysis. **Table S3**. Multiple sclerosis full data set. SNP rs number and minor allele are included, as well as the r^2^ values from the sensitivity analysis. The columns headed with the 1000 Genomes population codes are the allele frequencies for each SNP in those populations. The SNPs are listed in order of removal in the sensitivity analysis. **Table S4**. Height full data set. SNP rs number and minor allele are included, as well as the r^2^ values from the sensitivity analysis. The columns headed with the 1000 Genomes population codes are the allele frequencies for each SNP in those populations. The SNPs are listed in order of removal in the sensitivity analysis. **Table S5**. 1000 Genomes superpopulations, populations and population description. **Table S6**. PRS values for each population, before and after maximization. **Table S7**. Female height r^2^ values from the maximization analysis. **Table S8**. Lactase persistence filtered data set. **Table S9**. Melanoma filtered allele frequency data. **Table S10**. Multiple sclerosis filtered allele frequency data. **Table S11**. Male Height filtered allele frequency data. **Table S12**. Female Height filtered allele frequency data.

## Data Availability

All included data are publicly and freely available.
