# Supplementary figures and images for: Estimating prevalence of human traits among populations from polygenic risk scores
Source: Hum Genomics. 2021 Dec 13;15:70. doi: 10.1186/s40246-021-00370-z (PMC8670062; doi:10.1186/s40246-021-00370-z)

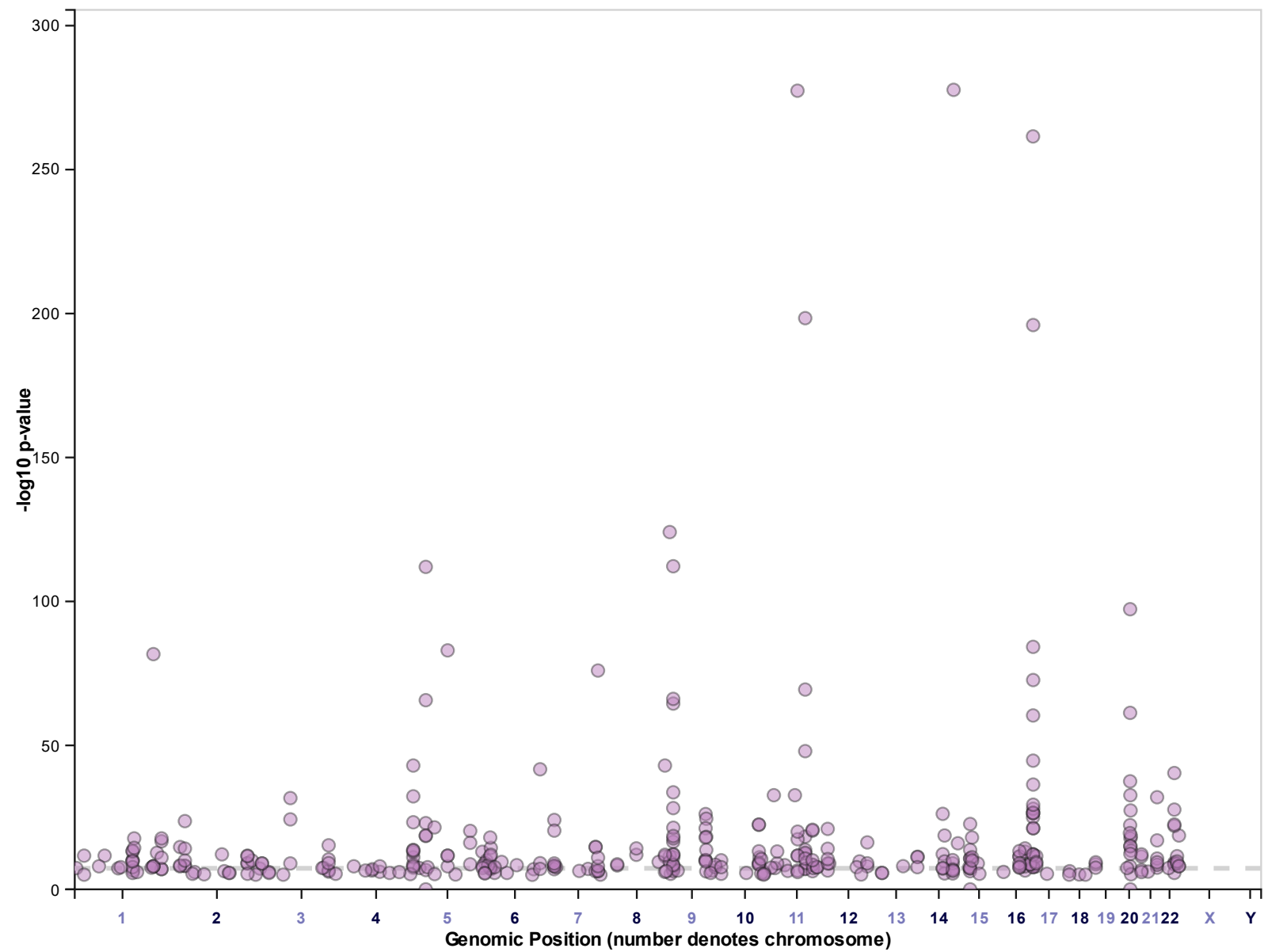

Supplement: Supplementary file 1 — Additional file 1: Figure S1. LocusZoom chromosomal location plot of the full melanoma SNP set. [file 40246_2021_370_MOESM1_ESM.pdf]

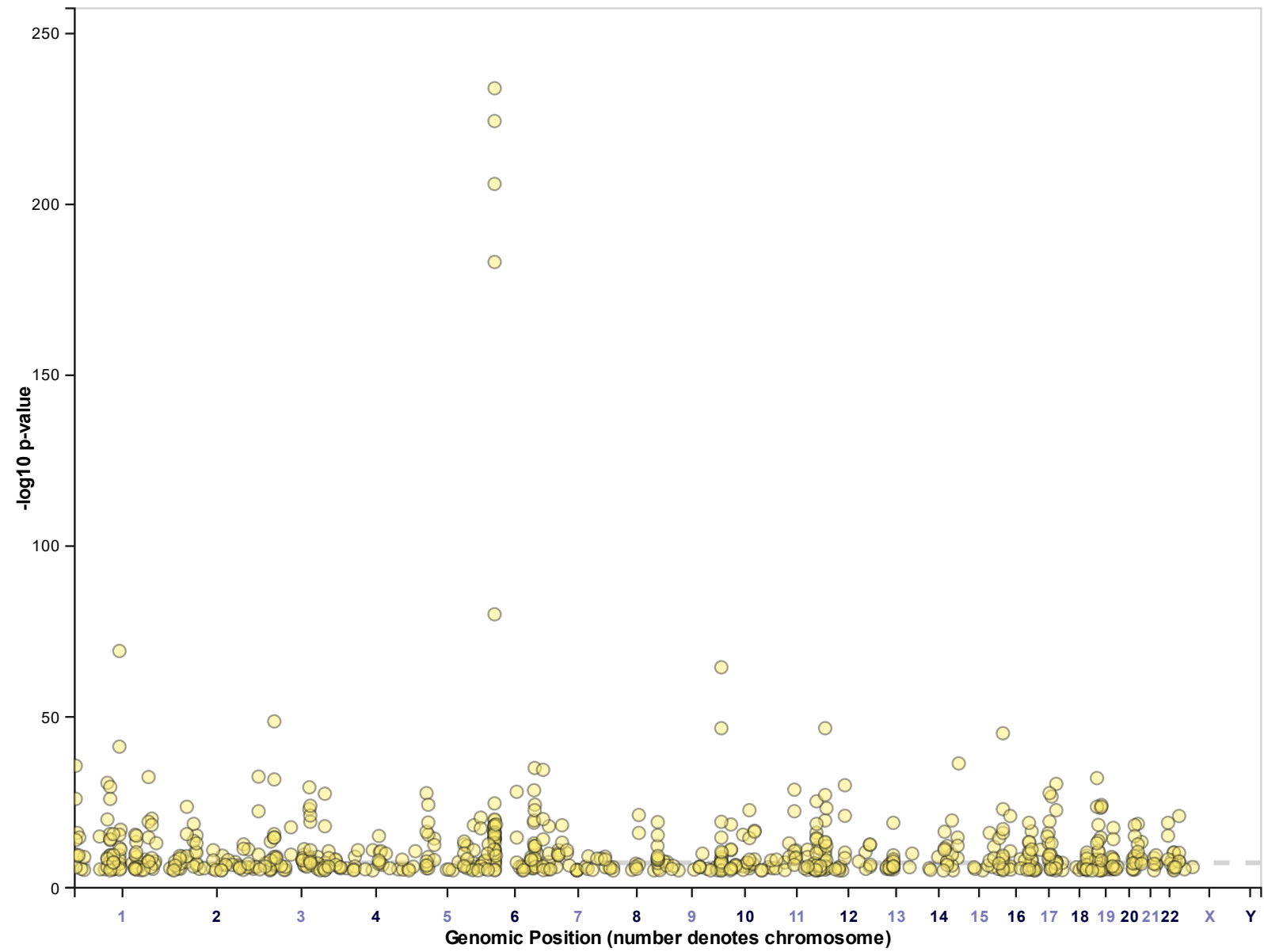

Supplement: Supplementary file 2 — Additional file 2: Figure S2. LocusZoom chromosomal location plot of the full multiple sclerosis SNP set. [file 40246_2021_370_MOESM2_ESM.pdf]

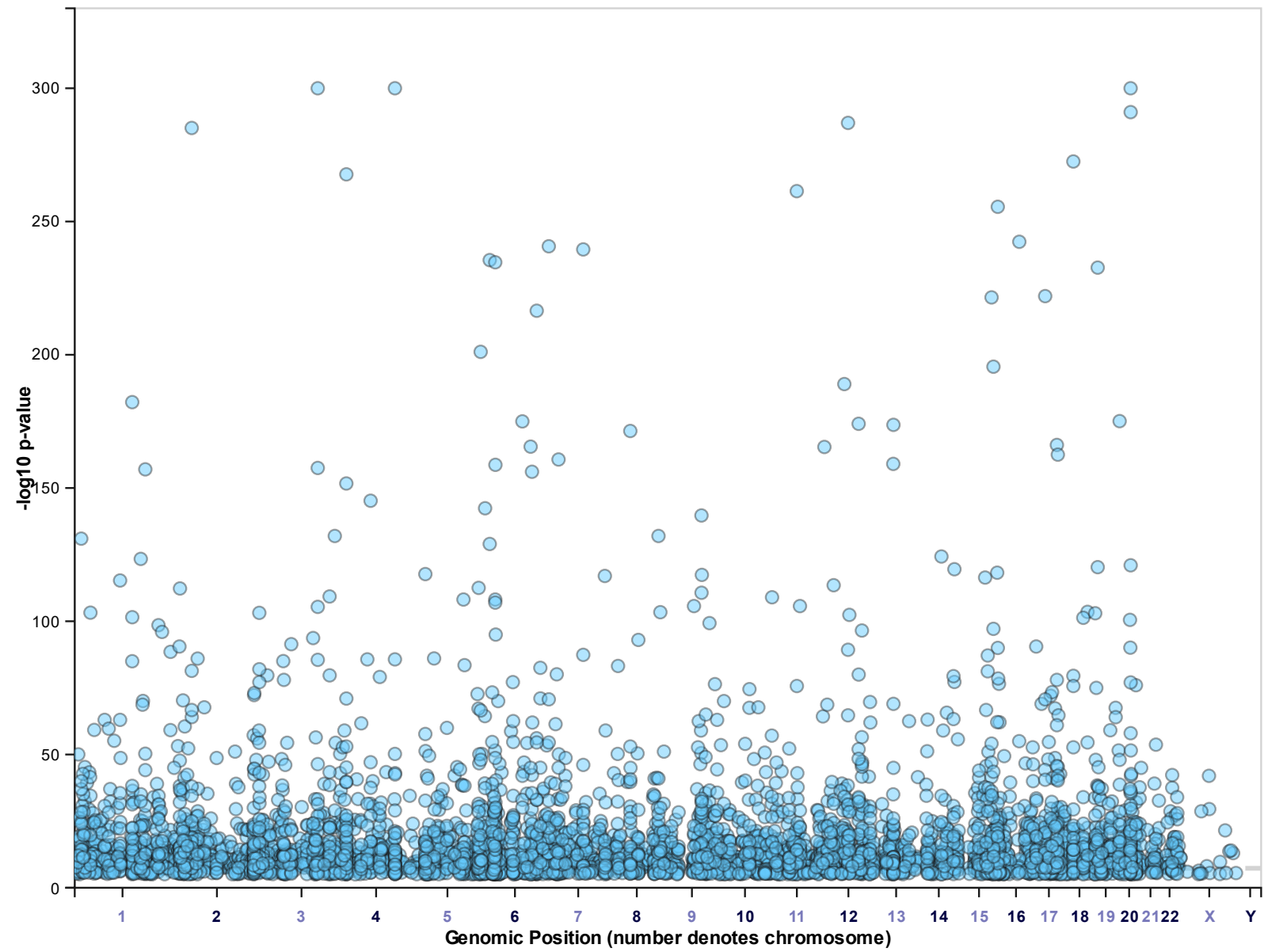

Supplement: Supplementary file 3 — Additional file 3: Figure S3. LocusZoom chromosomal location plot of the full height SNP set. [file 40246_2021_370_MOESM3_ESM.pdf]

A

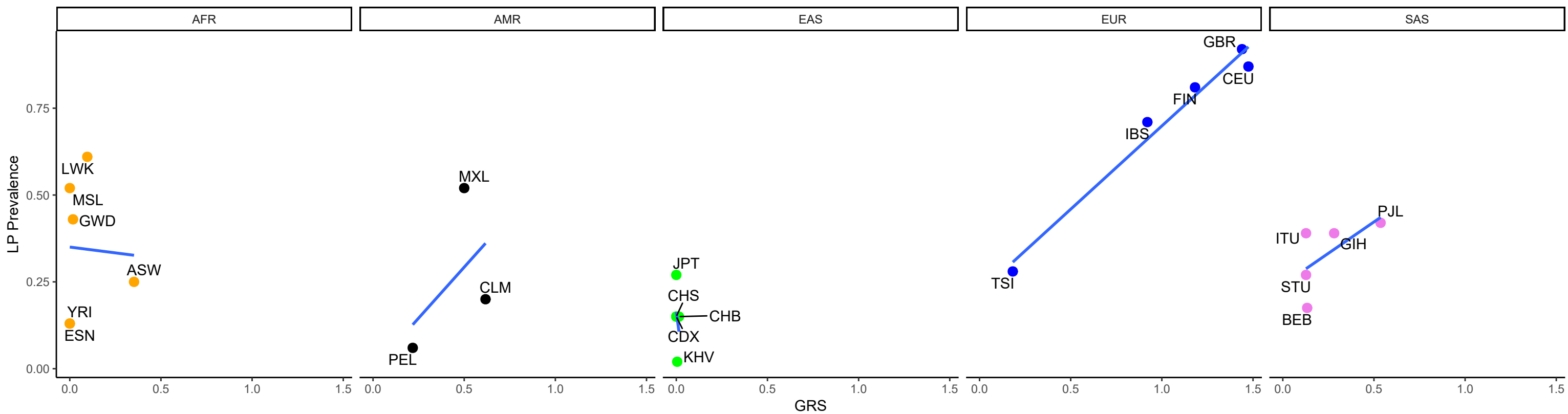

B

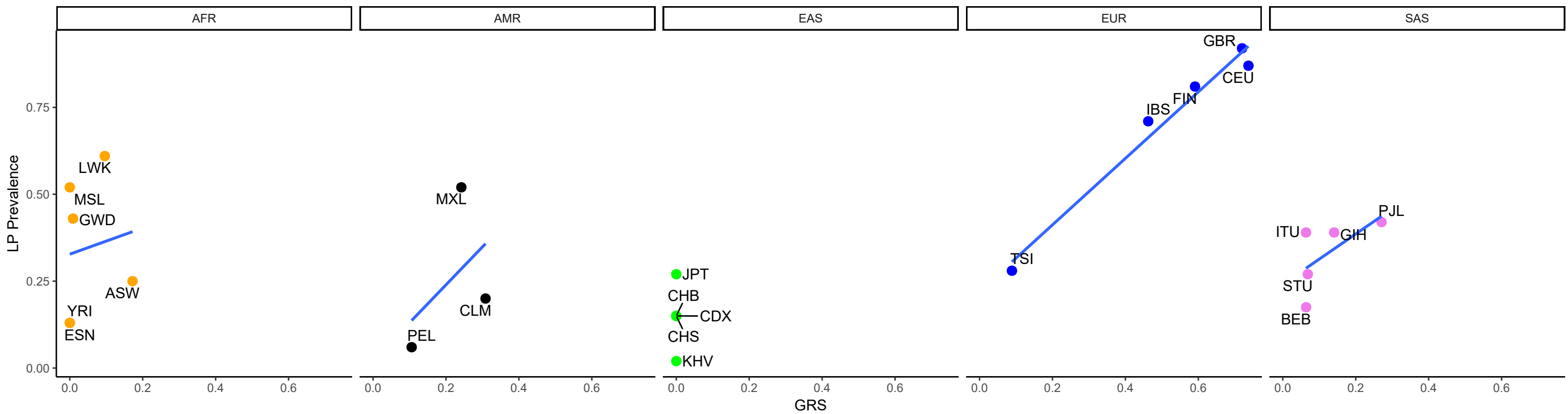

superpopulation ● AFR ● AMR ● EAS ● EUR ● SAS

Supplement: Supplementary file 4 — Additional file 4: Figure S4. Lactase persistence separated by super population. The data points are colored according to the super populations: AFR (orange), AMR (black), EAS (green), EUR (blue) and SAS (purple). A) Full model by super population: AFR (r2 = 0.0021, p-value: 0.9314), AMR (r2 = 0.2608, p-value: 0.659), EAS (r2 = 0.077, p-value: 0.6514), EUR (r2 = 0.9734, p-value: 0.00185) and SAS (r2 = 0.3847, p-value: 0.2643). B) Super populations after maximization: AFR (r2 = 0.0177, p-value: 0.8017), AMR (r2 = 0.2284, p-value: 0.683), EAS (no data), EUR (r2 = 0.9747, p-value: 0.00172) and SAS (r2 = 0.3914, p-value: 0.0580). [file 40246_2021_370_MOESM4_ESM.pdf]

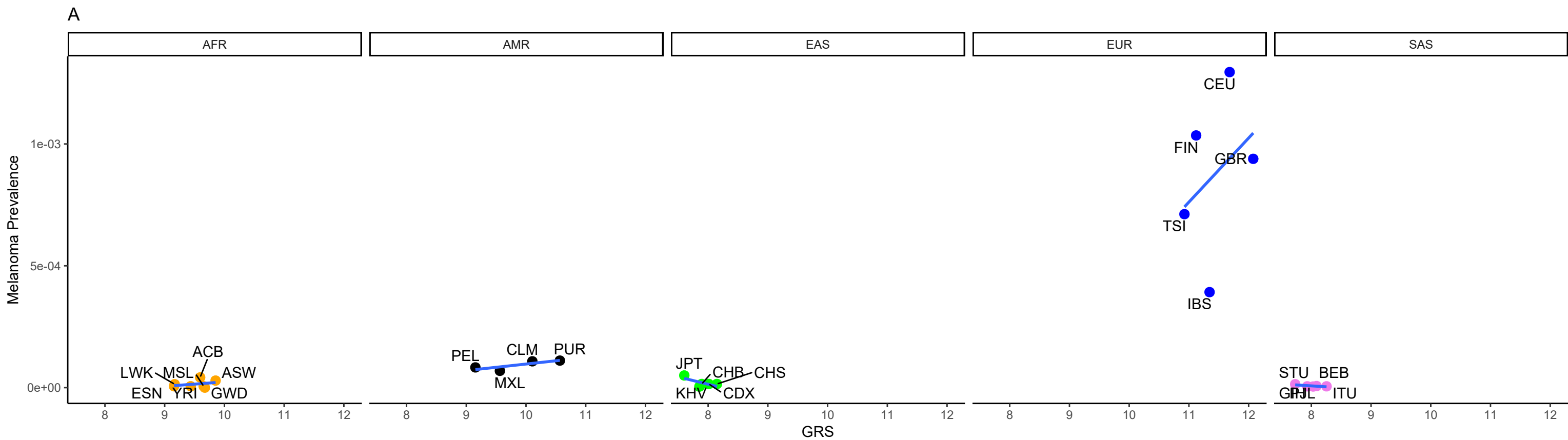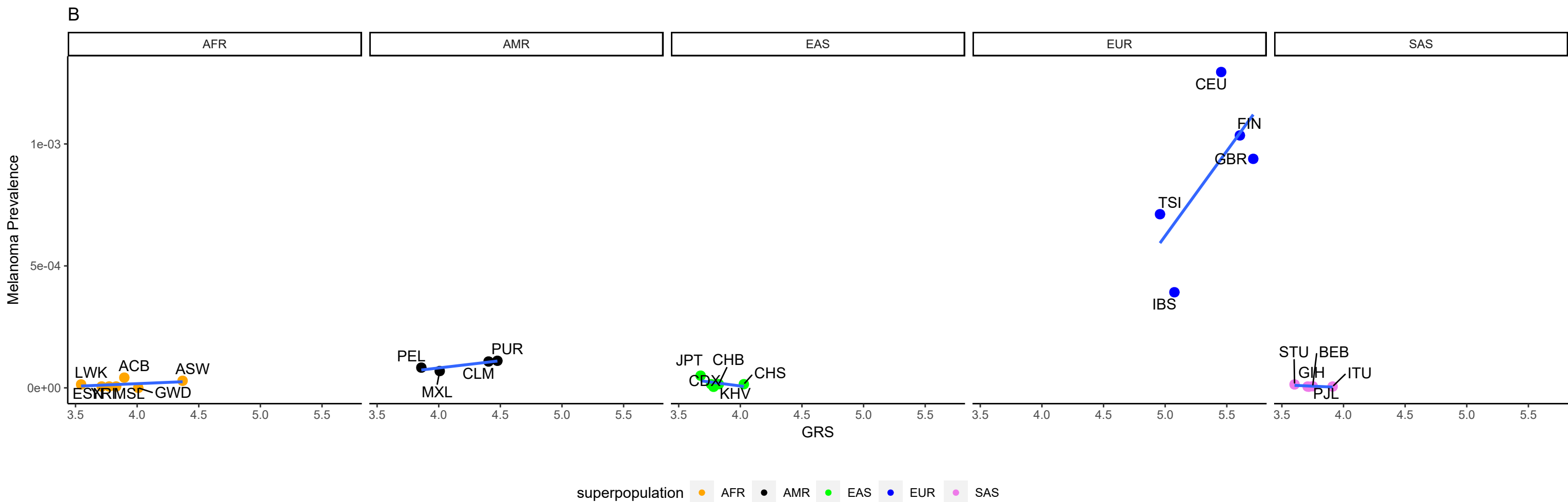

Supplement: Supplementary file 5 — Additional file 5: Figure S5. Melanoma separated by super population. The data points are colored according to the super populations: AFR (orange), AMR (black), EAS (green), EUR (blue) and SAS (purple). A) Full model by super population: AFR (r2 = 0.1178, p-value: 0.5718), AMR (r2 = 0.6664, p-value: 0.1837), EAS (r2 = 0.4958, p-value: 0.1844), EUR (r2 = 0.0421, p-value: 0.7949) and SAS (r2 = 0.5914, p-value: 0.1285). B) Super populations after maximization: AFR (r2 = 0.1767, p-value: 0.481), AMR (r2 = 0.7766, p-value: 0.1187), EAS (r2 = 0.2399, p-value: 0.4022), EUR (r2 = 0.6268, p-value: 0.2083) and SAS (r2 = 0.4324, p-value: 0.2278). [file 40246_2021_370_MOESM5_ESM.pdf]

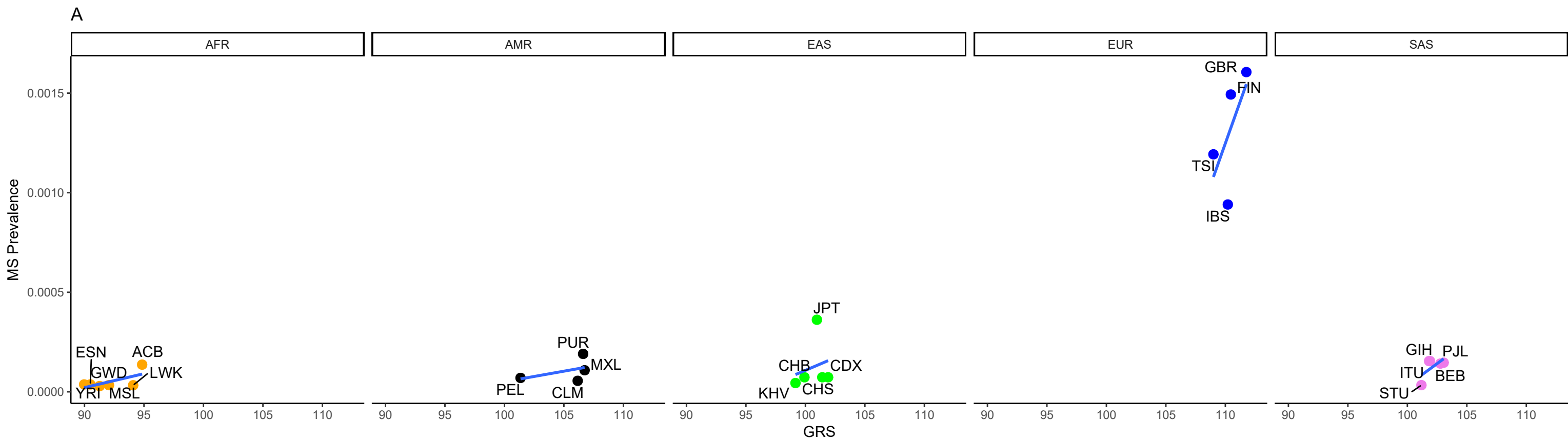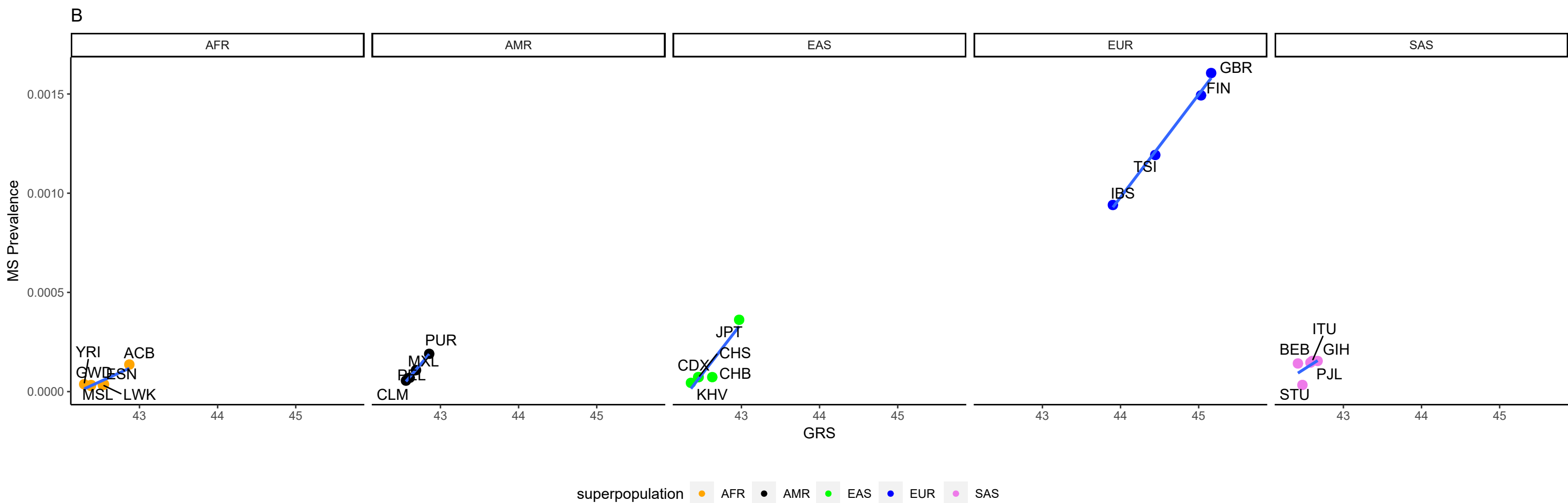

Supplement: Supplementary file 6 — Additional file 6: Figure S6. Multiple sclerosis separated by super population. The data points are colored according to the super populations: AFR (orange), AMR (black), EAS (green), EUR (blue) and SAS (purple). A) Full model super populations: AFR (r2 = 0.4336, p-value: 0.155), AMR (r2 = 0.1958, p-value: 0.5575), EAS (r2 = 0.0459, p-value: 0.7293), EUR (r2 = 0.3676, p-value: 0.3937) and SAS (r2 = 0.3775, p-value: 0.270). B) Super populations after maximization: AFR (r2 = 0.7781, p-value: 0.02003), AMR (r2 = 0.9821, p-value: 0.008995), EAS (r2 = 0.8775, p-value: 0.0189), EUR (r2 = 0.9988, p-value: 0.000617) and SAS (r2 = 0.2356, p-value: 0.407) . [file 40246_2021_370_MOESM6_ESM.pdf]

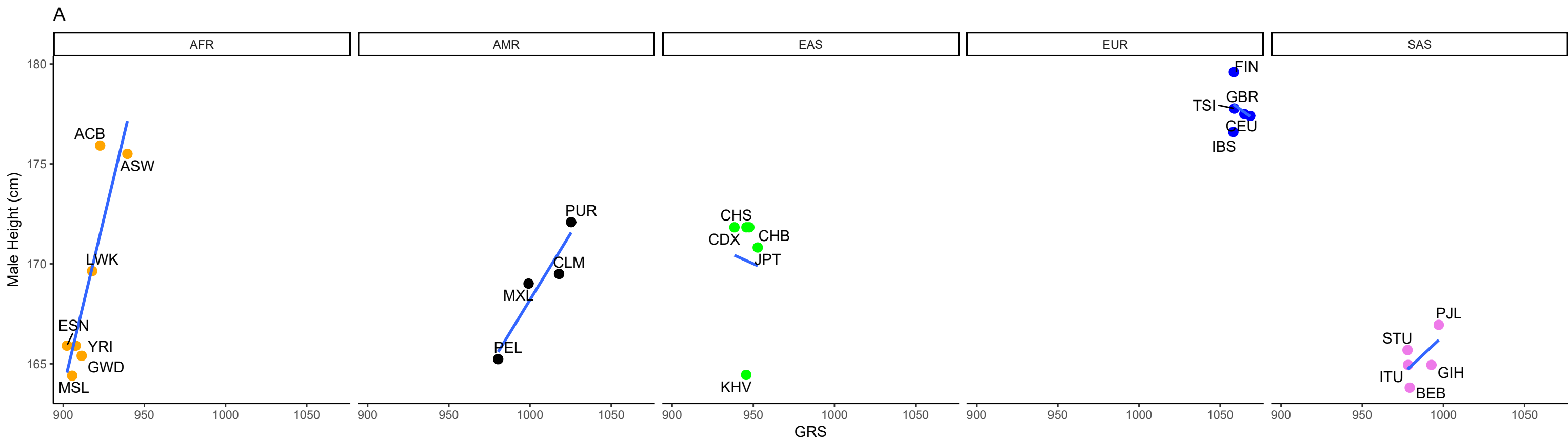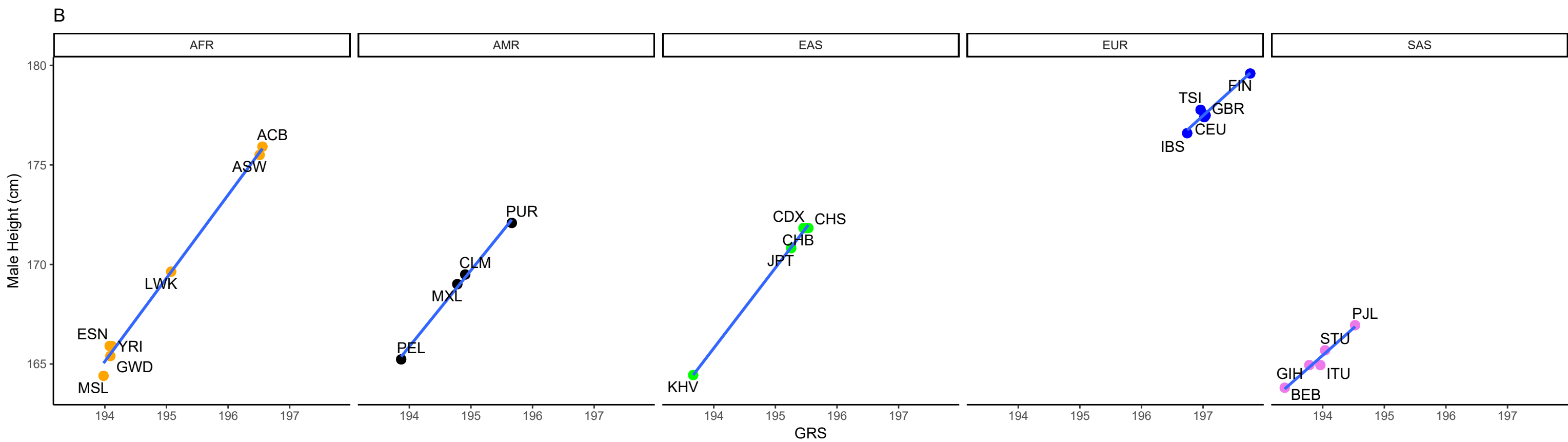

superpopulation

- AFR
- AMR
- EAS
- EUR
- SAS

Supplement: Supplementary file 7 — Additional file 7: Figure S7. Male height separated by super population. The data points are colored according to the super populations: AFR (orange), AMR (black), EAS (green), EUR (blue) and SAS (purple). A) Super populations with full model: AFR (r2 = 0.7835, p-value: 0.00806), AMR (r2 = 0.8628, p-value: 0.0522), EAS (r2 = 0.1003, p-value: 0.9254), EUR (r2 = 0.578, p-value: 0.551) and SAS: r2 = 0.1162, p-value: 0.2812. B) super populations after performing maximization: AFR (r2 = 0.5534, p-value: 6.549 x 10^-7^), AMR (r2 = 0.8556, p-value: 0.001876), EAS (r2 = 0.0163, p-value: 2.052 x 10^-5^), EUR (r2 = 0.9158, p-value: 0.01064) and SAS (r2 = 0.0475, p-value: 0.002888). [file 40246_2021_370_MOESM7_ESM.pdf]

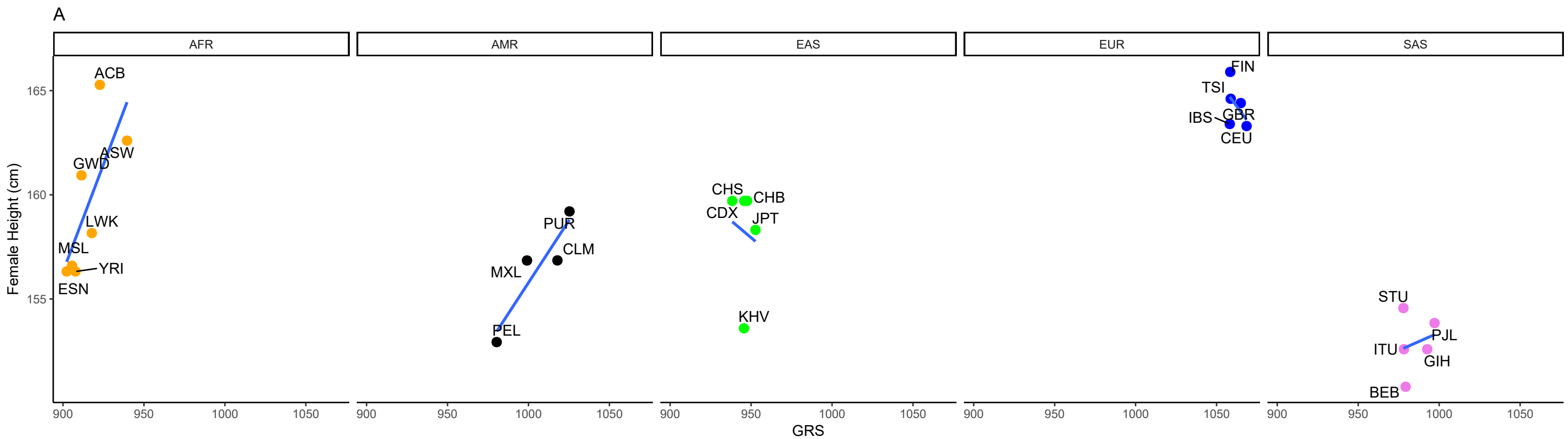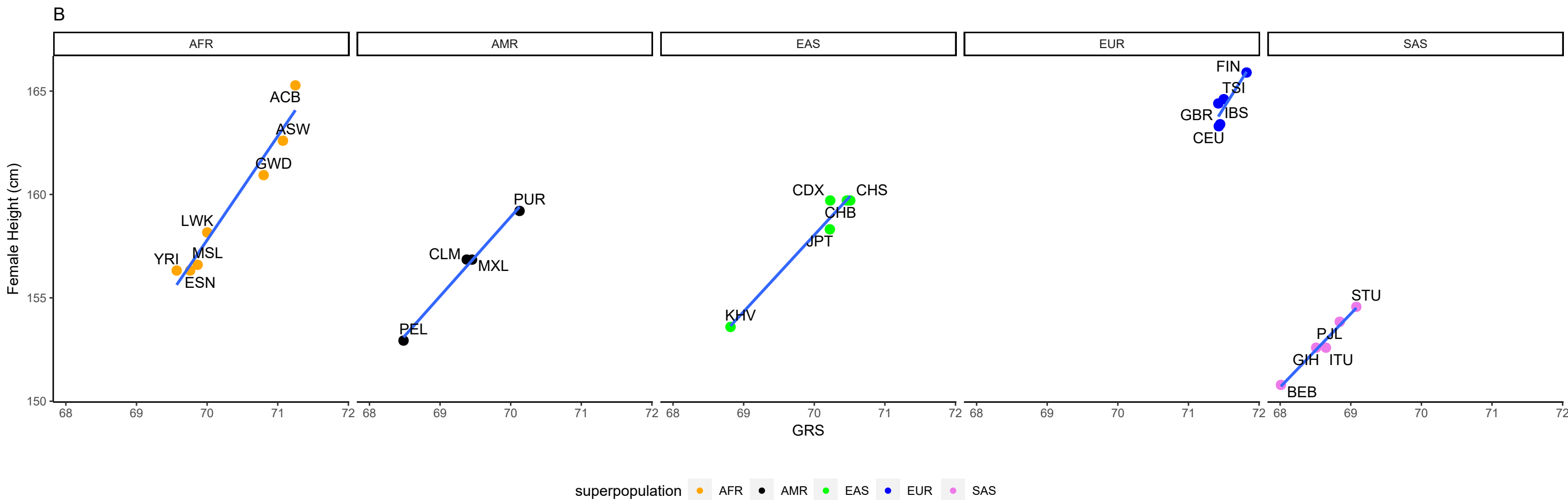

Supplement: Supplementary file 8 — Additional file 8: Figure S8. Female height separated by super population. The data points are colored according to the super populations: AFR (orange), AMR (black), EAS (green), EUR (blue) and SAS (purple). A) Super populations with full model: AFR (r2 = 0.5534, p-value: 0.05523), AMR (r2 = 0.8556, p-value: 0.07501), EAS (r2 = 0.0163, p-value: 0.8379), EUR (r2 = 0.2222, p-value: 0.4229) and SAS (r2 = 0.0475, p-value: 0.7246). B) Super populations after maximization: AFR (r2 = 0.9533, p-value: 0.0001627), AMR (r2 = 0.9917, p-value: 0.004174), EAS (r2 = 0.963, p-value: 0.003058), EUR (r2 = 0.754, p-value: 0.05619) and SAS (r2 = 0.9761, p-value: 0.001584). [file 40246_2021_370_MOESM8_ESM.pdf]

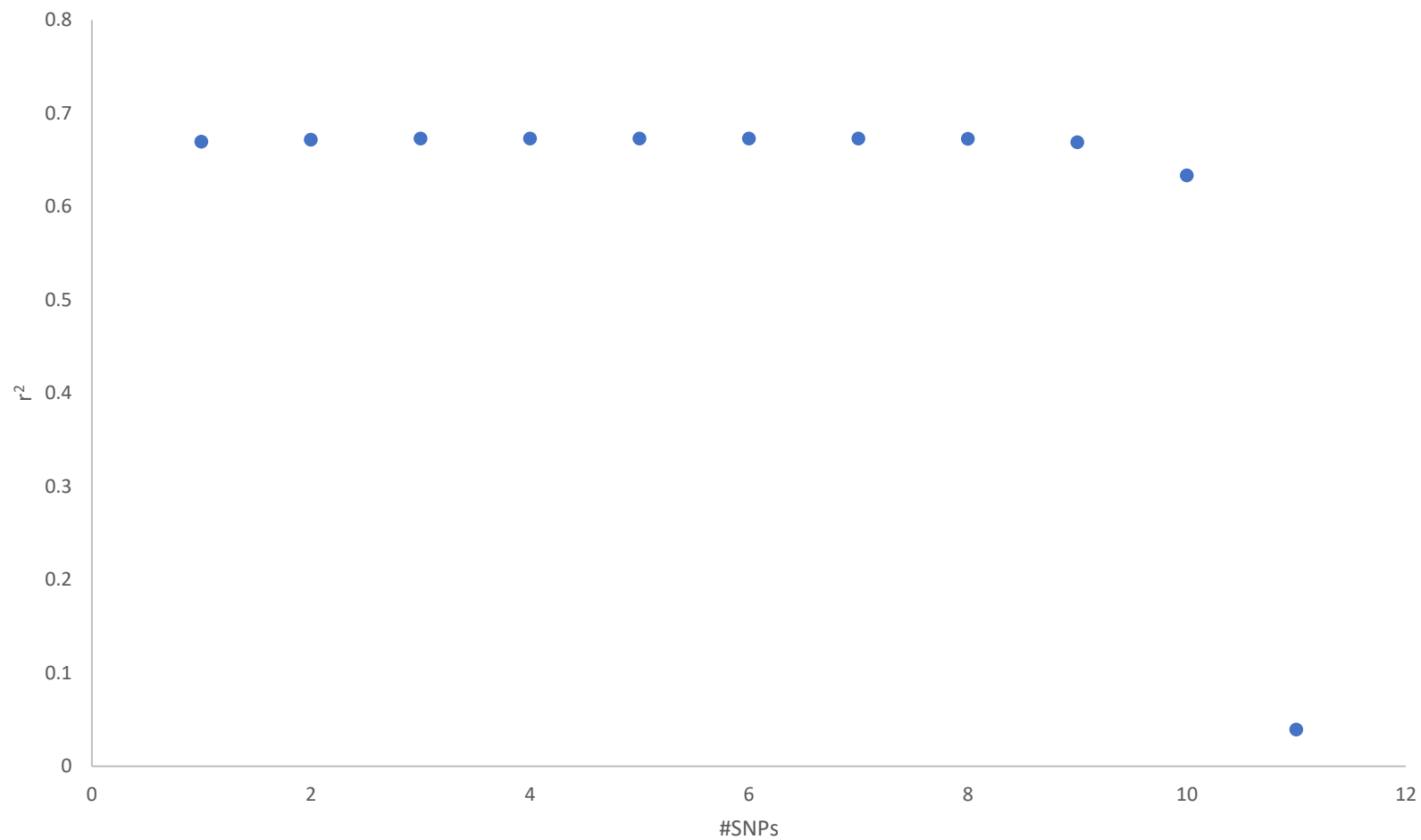

**Figure S9.** Lactase persistence  $r^2$  maximization.

Supplement: Supplementary file 9 — Additional file 9: Figure S9. Lactase persistence maximization analysis r2 values. [file 40246_2021_370_MOESM9_ESM.pdf]

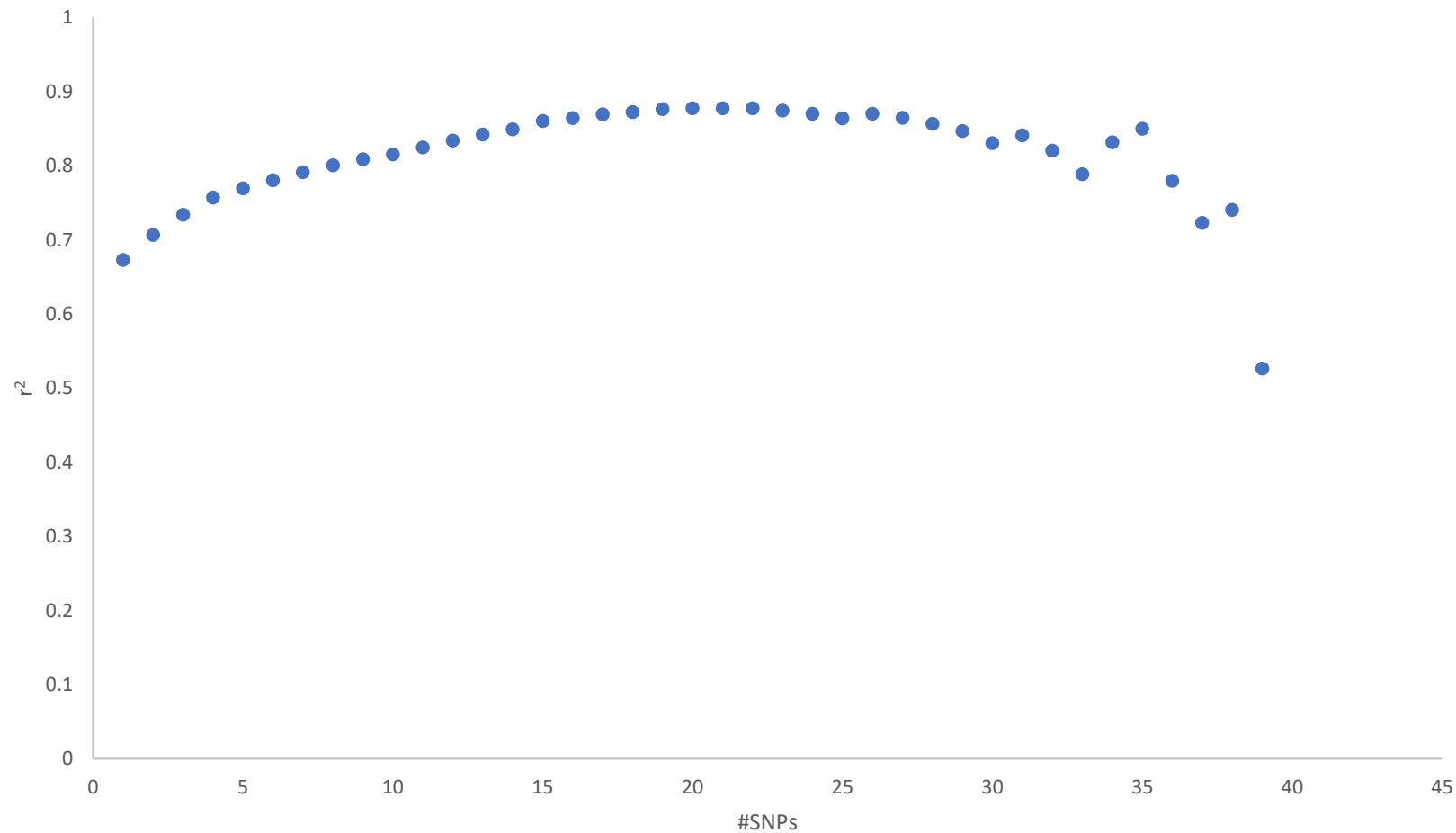

**Figure S10.** Melanoma  $r^2$  maximization.

Supplement: Supplementary file 10 — Additional file 10: Figure S10. Melanoma maximization analysis r2 values. [file 40246_2021_370_MOESM10_ESM.pdf]

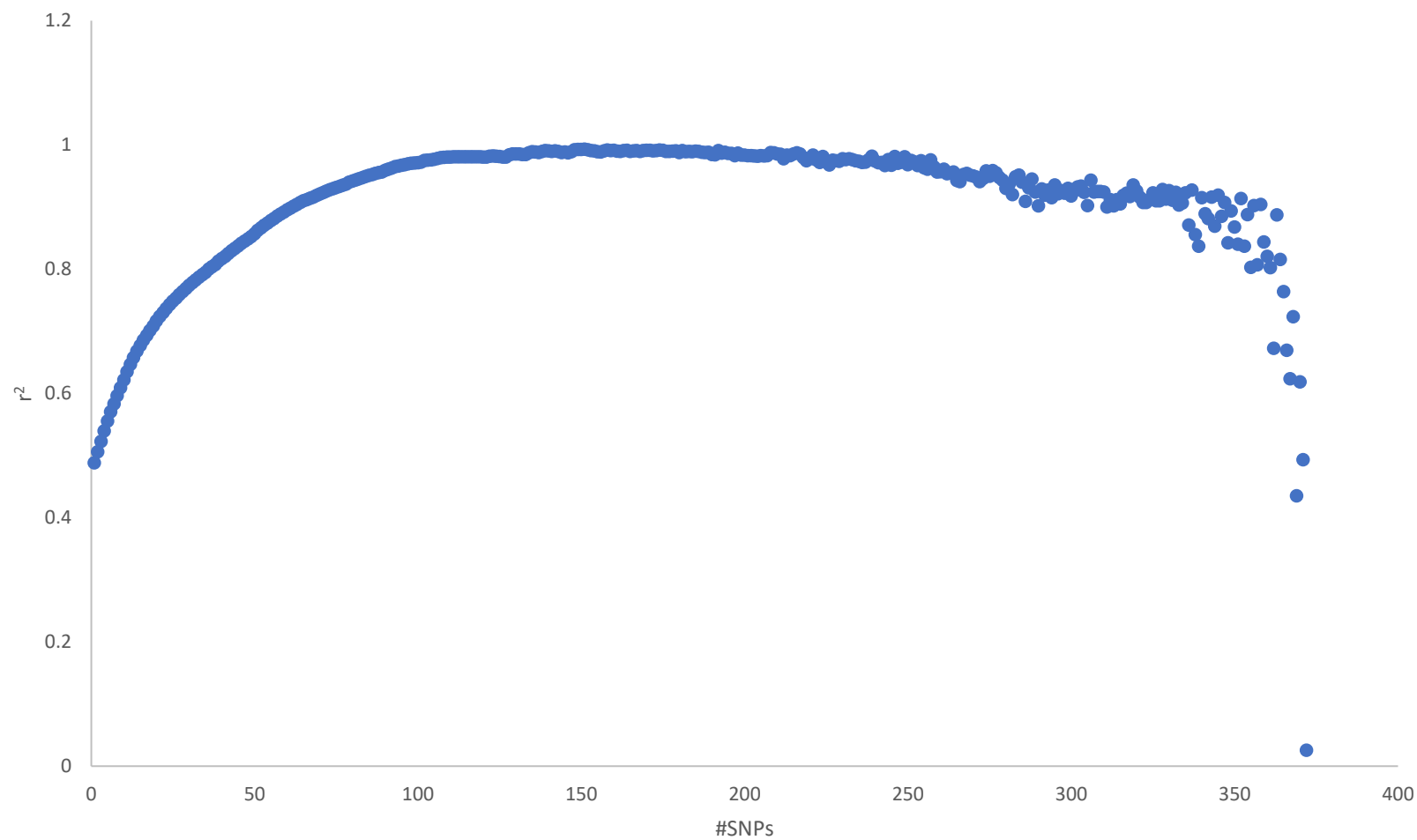

**Figure S11.** Multiple sclerosis  $r^2$  maximization.

Supplement: Supplementary file 11 — Additional file 11: Figure S11. Multiple sclerosis maximization analysis r2 values. [file 40246_2021_370_MOESM11_ESM.pdf]

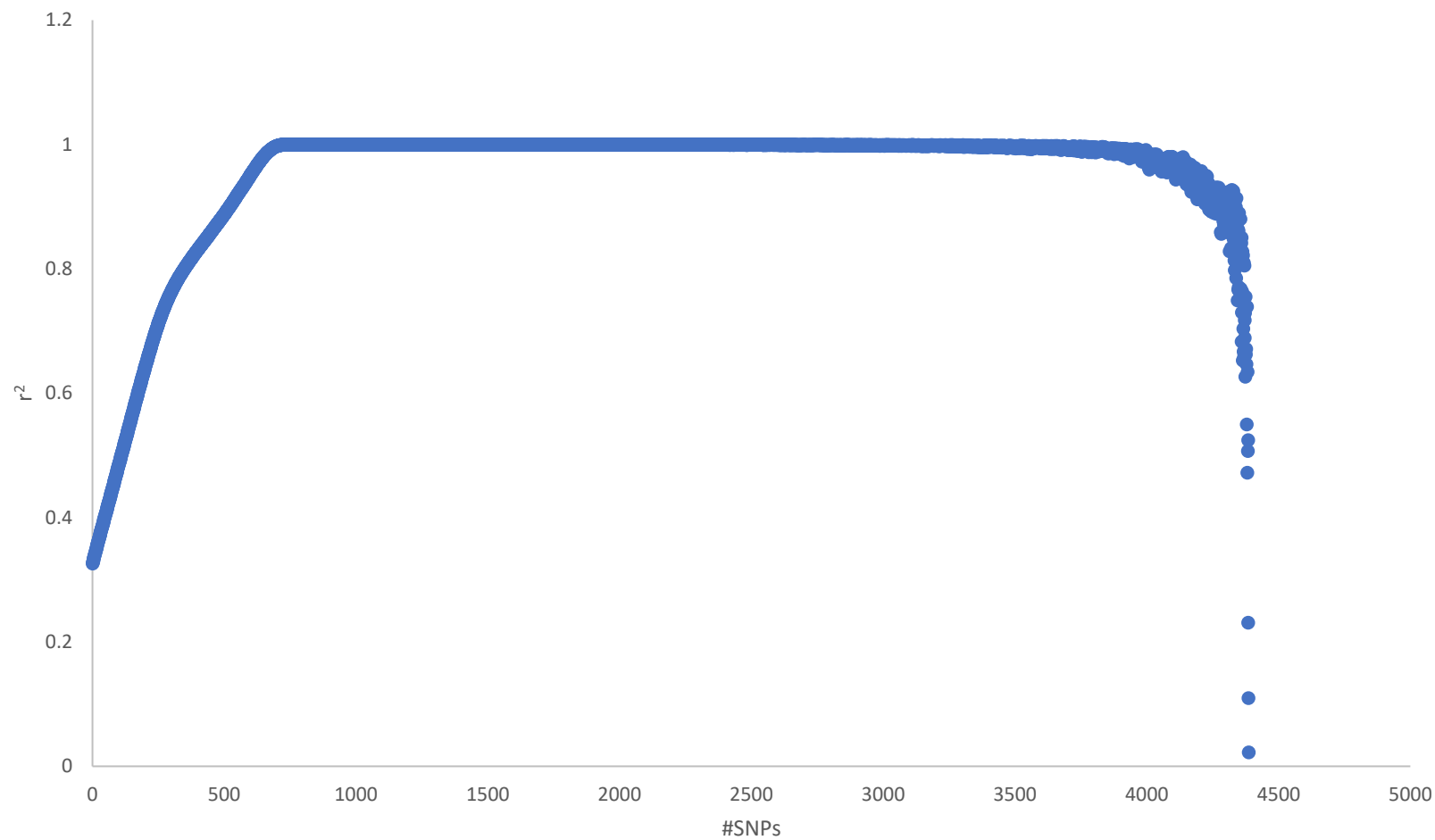

**Figure S12.** Male height  $r^2$  maximization.

Supplement: Supplementary file 12 — Additional file 12: Figure S12. Male height maximization analysis r2 values. [file 40246_2021_370_MOESM12_ESM.pdf]

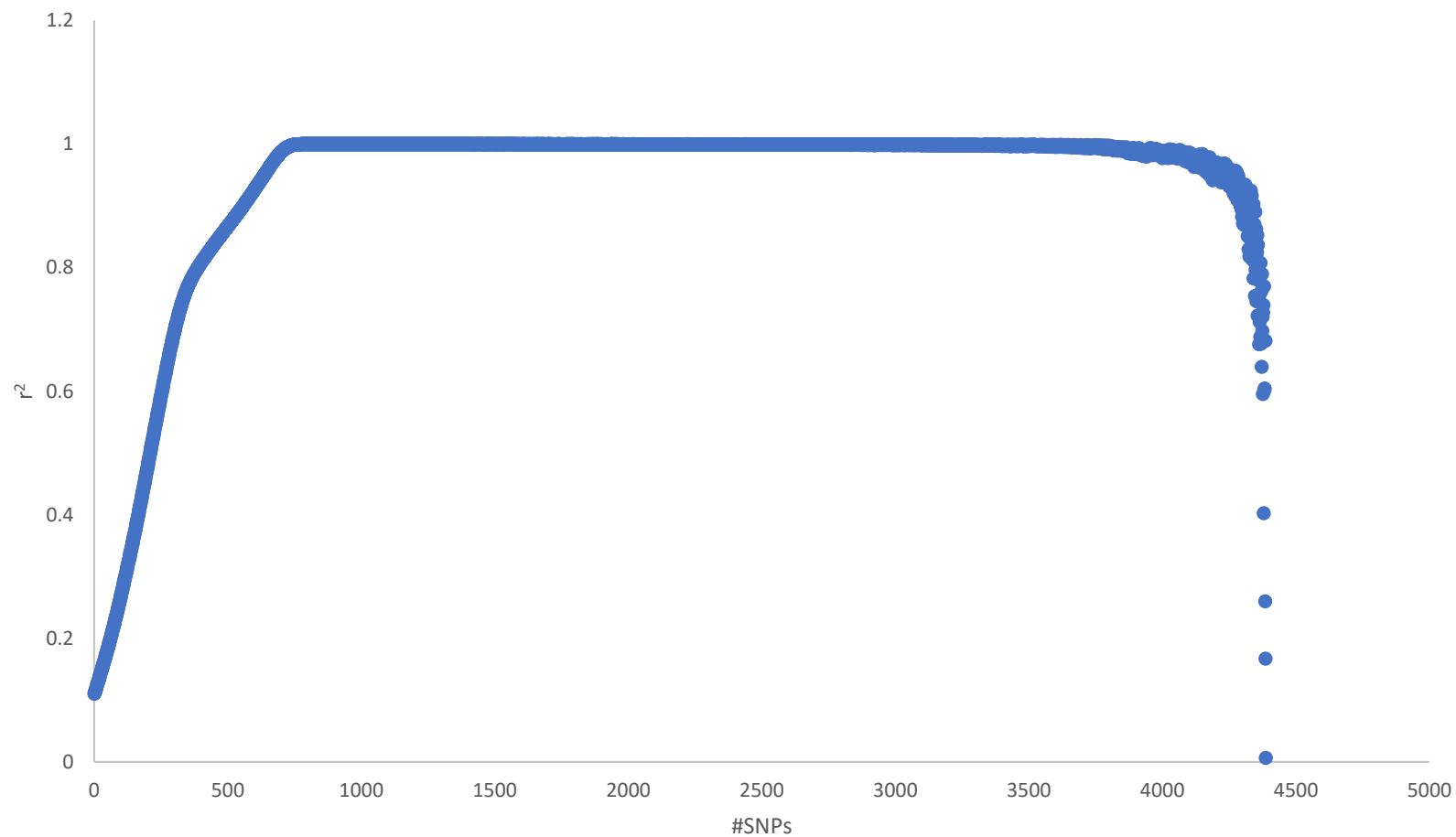

**Figure S13.** Female height  $r^2$  maximization.

Supplement: Supplementary file 13 — Additional file 13: Figure S13. Female height maximization analysis r2 values. [file 40246_2021_370_MOESM13_ESM.pdf]
